# Supplementary material for: Healthy Eating and Active Lifestyles for Diabetes (HEAL-D): study protocol for the design and feasibility trial, with process evaluation, of a culturally tailored diabetes self-management programme for African-Caribbean communities
Source: BMJ Open. 2019 Mar 1;9(2):e023733. doi: 10.1136/bmjopen-2018-023733 (PMC6398623; doi:10.1136/bmjopen-2018-023733)
Supplement: Author's manuscript [file bmjopen-2018-023733.draft_revisions.pdf]

BMJ Open is committed to open peer review. As part of this commitment we make the peer review history of every article we publish publicly available.

When an article is published we post the peer reviewers' comments and the authors' responses online. We also post the versions of the paper that were used during peer review. These are the versions that the peer review comments apply to.

The versions of the paper that follow are the versions that were submitted during the peer review process. They are not the versions of record or the final published versions. They should not be cited or distributed as the published version of this manuscript.

BMJ Open is an open access journal and the full, final, typeset and author-corrected version of record of the manuscript is available on our site with no access controls, subscription charges or pay-per-view fees (http://bmjopen.bmj.com).

If you have any questions on BMJ Open's open peer review process please email editorial.bmjopen@bmj.com

BMJ Open

Study Protocol: Healthy Eating and Active Lifestyles for Diabetes (HEAL-D) - a feasibility trial, with process evaluation, of a culturally-tailored diabetes self-management programme for African-Caribbean communities

Journal:	*BMJ Open*
Manuscript ID	bmjopen-2018-023733
Article Type:	Protocol
Date Submitted by the Author:	20-Apr-2018
Complete List of Authors:	Goff, Louise; Kings College Lonodn, Diabetes and Nutritional Sciences<br>Moore, Amanda; Kings College Lonodn, Diabetes and Nutritional Sciences<br>Rivas, Carol; University of Southamptom, Faculty of Health Sciences<br>Harding, Seeromanie; King\'s College London Division of Diabetes and Nutritional Sciences, Diabetes & Nutritional Sciences Division
Keywords:	type 2 diabetes, ethnicity, culture, diet, lifestyle, education

SCHOLARONE™
Manuscripts

For peer review only

Study Protocol: Healthy Eating and Active Lifestyles for Diabetes (HEAL-D) - a feasibility trial, with process evaluation, of a culturally-tailored diabetes self-management programme for African-Caribbean communities

Louise M. Goff[1#], Amanda P. Moore[1], Carol Rivas[2], Seeromanie Harding[1]

[1]King's College London, Departments of Diabetes & Nutritional Sciences, School of Life Course Sciences, Faculty of Life Sciences & Medicine, [2]Institute of Education, University College London

[#]Corresponding author: Dr Louise M. Goff, Diabetes Research Group, King's College London, Henriette Raphael Building, Room 2.29, Guy's Campus, King's College London, London SE1 1UL. T: 020 7848 6111; E: louise.goff@kcl.ac.uk

For peer review only

For peer review only - http://bmjopen.bmj.com/site/about/guidelines.xhtml

ABSTRACT

Introduction

Black-British communities are disproportionately burdened by type 2 diabetes (T2D); it occurs earlier, with worse control at diagnosis, and is associated with poorer outcomes compared to White-British groups. Tackling these inequalities is a priority for both healthcare providers and patients. Culturally-tailored diabetes education provides long-term benefits superior to standard care but to date such programmes have only been developed in the USA. The Healthy Eating and Active Lifestyles for Diabetes (HEAL-D) programme of research aims to develop a culturally-tailored, evidence-based diet and lifestyle intervention for managing T2D in Black-British communities; and to evaluate its acceptability and the feasibility of conducting a future effectiveness trial of HEAL-D.

Methods & analysis

Informed by MRC Complex Interventions guidance this research will rigorously develop and evaluate the implementation of the HEAL-D intervention. In Phase 1 the intervention will be developed through co-design methods, which will seek to foster community engagement and identify the intervention's underpinning programme theory and cultural adaptations. Focus groups and interviews will be conducted with key stakeholders (patients, healthcare professionals and community leaders). The qualitative data will be analysed using the framework approach to identify priority behaviours of focus for the intervention, key barriers and facilitators to behaviour change and healthcare engagement, favoured settings, and a rudimentary draft of the cultural adaptations. We will map our analysis onto the Capability-Opportunity-Motivation-Behaviour (COM-B) framework from the Behaviour Change Wheel to ascertain appropriate behaviour change techniques for the intervention. In Phase 2 process evaluation methods will evaluate the delivery and acceptability of HEAL-D, and the feasibility of conducting a future effectiveness trial.

For peer review only - http://bmjopen.bmj.com/site/about/guidelines.xhtml

Ethics & dissemination

This study is funded by a National Institute of Health Research Fellowship (CDF-2015-08-006). It has been approved by the Fulham: London Research Ethics Committee (17-LO-1954). Results will be disseminated at national and international conferences, in peer-reviewed publications and through local and national clinical diabetes networks.

Strengths and limitations of this study

- This study employs rigorous complex intervention methodology to develop and evaluate the implementation of a culturally-tailored diabetes self-management intervention.
- Our intervention, HEAL-D, is designed using a 'bottom-up' approach, employing participatory co-design methods to foster community engagement and partnership.
- We will identify the cultural adaptations of our intervention and its underpinning theoretical basis through thematic analysis and the COM-B behavior change framework.
- The feasibility study will provide us with key information about the feasibility of running a full-scale trial of HEAL-D.
- Process evaluation methods will enable us to understand how and why the intervention was effective or ineffective.

Keywords: African, Caribbean, ethnicity, type 2 diabetes, education, self-management, diet, lifestyle

For peer review only - http://bmjopen.bmj.com/site/about/guidelines.xhtml

INTRODUCTION

Type 2 diabetes (T2D) affects approximately 3 million people in England and consumes around 10% of the National Health Service (NHS) budget, estimated at almost £9 billion in 2011 (1). Diabetes and its associated complications place an illness burden on patients and carers, which disproportionately affects those from ethnic minority backgrounds (2). The estimated prevalence of T2D is up to 3 times higher for Black-British communities compared to White Europeans (3). T2D occurs at a younger age in Black-British people, with worse control at the time of diagnosis, and is associated with poorer outcomes (4, 5) thus making healthcare for this community a priority (6, 7).

Poor access to diabetes healthcare is a significant issue for minority ethnic groups (2). First-line diabetes management is situated in primary care and aims to promote patient involvement and self-management (8), enabling patients to adopt a healthy lifestyle and to manage their diabetes through support and education (9). To achieve this, diabetes services principally aim to deliver care that is patient-centred and intended to be responsive to individual culture, lifestyle and religion (10). However ethnic minority groups report finding it more difficult to access primary care services (11) and are more likely to report that they have not had the opportunity to attend a diabetes education course than White populations (12). Specifically, African-Caribbean (AfC) communities often report a distrust of medical advice and a desire for natural, non-pharmacological therapies (13). Furthermore, healthcare professionals are perceived as lacking cultural understanding (14) and their advice as lacking cultural relevance (15) or being poorly adapted to culture and needs (13) despite their intentions; these issues may contribute to the poorer diabetes outcomes and increased morbidity experienced by AfC patients.

For peer review only - http://bmjopen.bmj.com/site/about/guidelines.xhtml

Culturally tailored healthcare is proposed to be one of the main ways in which healthcare disparities can be addressed (16-18) and is identified as a priority by patients (6). Culturally-tailored diabetes education has demonstrated greater improvements in diabetes control and knowledge than usual care, and the benefits are maintained long-term (17, 19). However, to date, culturally tailored interventions for the African diaspora have largely been based in the USA, and may not translate to UK AfC communities or healthcare structures (18).

Healthy Eating & Active Lifestyles for Diabetes (HEAL-D) is a two-phase programme of research in which a culturally-tailored, evidence-based diet and lifestyle intervention for managing T2D in African and Caribbean communities will be developed using co-design methods, and subsequently evaluated in a feasibility trial. The purpose of this article is to present the protocol for HEAL-D.

For peer review only

PURPOSE & AIMS

The overall aims of this research are to develop a culturally-tailored, evidence-based diet and lifestyle intervention for managing T2D among AfC communities in primary care, called HEAL-D, and to determine the feasibility of evaluating HEAL-D through a future effectiveness trial.

The objectives are to:

1. Develop a diet and lifestyle intervention appropriately tailored for AfC patients through co-design methods.
2. Identify effective modes of sustainable engagement of key stakeholders, including patients, healthcare providers, and community leaders.
3. Establish the feasibility of embedding delivery of a culturally-tailored programme into existing care pathways.
4. Establish the feasibility of conducting an effectiveness trial of HEAL-D, considering issues such as participation rates and reach, potential effect sizes and cost evaluation.

For peer review only - http://bmjopen.bmj.com/site/about/guidelines.xhtml

METHODS AND ANALYSIS

Guided by the MRC Complex Interventions framework (20), HEAL-D will consist of two distinct phases: phase 1 is a formative phase in which the intervention will be developed; and in phase 2, the intervention will be evaluated in a feasibility trial (Figure 1).

Phase 1 – Development of a culturally-tailored diet and lifestyle intervention for managing T2D

The HEAL-D intervention aims to provide a programme of self-management education and behaviour change for AfC patients with T2D to achieve existing evidence-based diet and lifestyle goals (21), specifically:

1. Modest weight loss (5-10%) or weight maintenance in those of healthy weight
2. 150 minutes per week of moderate-to-vigorous intensity aerobic physical activity plus 2 sessions per week of strength training
3. Balanced carbohydrate intakes through portion control and promotion of low glycaemic index and wholegrain sources
4. Limited saturated fat intake (<10% of energy intake) and replacement with mono-unsaturated fats
5. Limited salt intake (<6g per day)
6. Oily fish consumption at least twice per week

Identifying the intervention's theoretical basis

Behavioural interventions are more effective if they have a theoretical under-pinning (20) (REF NICE 2014) so that what changes are expected and how these will be achieved can be predicted from consideration of known behaviour change techniques. The theoretical basis (or programme theory) for HEAL-D will be identified through two processes; firstly an evidence synthesis of key themes from published literature relating to adapting health

For peer review only - http://bmjopen.bmj.com/site/about/guidelines.xhtml

promotion interventions for ethnic minority groups, and secondly through new primary research. A number of recent systematic reviews have evaluated the evidence for designing health promotion interventions for ethnic minority groups. Aside from acknowledging the lack of UK-based studies, these reviews have made the following recommendations:

- Focus on community-level interventions rather than traditional ‘medical model’, individual-centred behavioural approaches (18).
- Employ participatory methods e.g. patient involvement in intervention design, lay-led delivery of interventions, and community empowerment. These are highly effective at improving health behaviours and self-efficacy across a number of conditions (22).
- Use community gathering places e.g. faith institutions, which offer the benefit of cultural relevancy and may reach populations who would not normally access self-management education (23).
- Foster community engagement to overcome issues of deep-rooted, historical distrust of medical advice and settings, develop and nurture trust between the researchers and community, and nurture the strong sense of collectivism and kinship networks that are evident amongst AfC communities.
- Acknowledge the powerful influence of social networks on health beliefs and behaviours (24); delivering care in a social context has been shown to promote engagement and be more effective than traditional individual-centred behavioural approaches (18).

HEAL-D will be grounded in these principles however, the theoretical basis of our intervention will be expanded through our co-design work.

Co-designing the intervention through participatory methods

For peer review only - http://bmjopen.bmj.com/site/about/guidelines.xhtml

HEAL-D will use participatory co-design methods to engage patients, healthcare providers and community leaders (e.g. church leaders, community group leads), in focus groups, interviews and workshops to achieve the following aims:

1) understand the issues that affect healthcare engagement and delivery for AfC T2D patients and thus contribute to the programme theory for our intervention, specifically (along with the evidence synthesis findings):
   - What are the barriers and facilitators to motivate and sustain changes in lifestyle behaviours in AfC T2D patients?
   - What barriers and enablers exist relating to embedding/implementation of a culturally-tailored intervention in existing care pathways?
2) design the cultural adaptations of our intervention:
   - What adaptations are needed to diet and lifestyle interventions for T2D to ensure cultural sensitisation?
3) foster community engagement and support for HEAL-D, thus facilitating implementation of the intervention:
   - What are the processes that effectively engage AfC communities (e.g. churches, community organisations) in a partnership to develop and deliver a lifestyle intervention for T2D management?

Focus groups and interviews

Focus groups, 8-10 groups of 6-8 participants, will be conducted with patients with T2D of AfC ethnicity, recruited through local churches, mosques and community groups, as well as through GP practices in Lambeth and Southwark. The focus groups will be conducted in local accessible community venues e.g. church hall, library, community centre. Patients will be purposively sampled to get a spread of socio-economic position, generational status and

For peer review only - http://bmjopen.bmj.com/site/about/guidelines.xhtml

ancestral origins, as principal factors impacting on health status, healthcare access and cultural behaviours in these groups (25-27). Separate focus groups will be conducted with men and women, and patients of direct African *vs* Caribbean ancestry, as they report different cultural barriers/facilitators to lifestyle change (25, 26). A topic guide (Figure 3) based on themes identified in the literature, will be used to steer discussions and ensure coverage of key themes whilst encouraging free discussion of opinion/perspective. Focus groups are being used to understand normative needs, as suited to the development of a community intervention.

Semi-structured interviews will be conducted with 8-10 healthcare providers, including general practitioners, practice nurses, diabetes specialist nurses, diabetes specialist dietitians and commissioners. The interviews will cover issues relating to healthcare needs and engagement of AfC patients, experiences of delivering healthcare to AfC patients, and barriers and facilitators to working in partnership with community groups to deliver care for AfC communities (Figure 3). Interviews have been selected for this part of the study to enable us to gather a full range of experiences and therefore optimise implementation.

Community leaders representing faith and non-faith institutions (n=4-6) will be invited to participate in semi-structured interviews. Leaders will be identified initially through existing networks e.g. Diabetes UK Community Champions initiative. Word-of-mouth and 'snow-balling', techniques that are highly effective within these communities, will be used to recruit a wider network. The interviews will cover issues relating to the role of community networks in promoting health of AfC communities, sustaining health amongst community members, and opportunities for greater impact (Figure 3).

Analysis

For peer review only

The focus groups and interviews will be digitally recorded and transcribed verbatim. The data will be analysed using the framework approach in NVivo (QSR International), theoretically driven by socio-ecological theory to identify themes relating to issues at the individual, family, community and healthcare delivery levels and how these influence self-efficacy and behaviour change. Our analysis will identify priority behaviours of focus for the intervention, key barriers and facilitators to behaviour change and healthcare engagement, favoured settings, and a rudimentary draft of the cultural adaptations. Deviant case analysis, that is consideration of cases that do not fit the general picture, will be undertaken, though our primary interest is in the commonalities as this is a community level intervention. Primary coding and development of a coding scheme will be carried out by a single researcher; a second researcher will independently use this coding scheme to code 20% of the data for cross-comparison, to improve dependability. This will provide methodological rigour required for confidence in the analysis of the qualitative data. The themes will be fed-back and discussed with a Service User Group that will consist of representatives of patients, healthcare providers, and community leaders to ensure trustworthiness of conceptualisations.

We will divide our data into behavioural 'barriers' and 'facilitators' where possible. To ascertain appropriate behaviour change techniques for our intervention (28) we will map our analysis onto the Capability-Opportunity-Motivation-Behaviour (COM-B) framework from the Behaviour Change Wheel (29), and thence in each case consider the outcome behaviours that our intervention will aim to achieve, a worked example is shown in Figure 2. We will use the COM-B framework to identify appropriate *functions* of our intervention to optimise enablers and overcome barriers to achievement of planned outcomes, e.g. 'education' for capability barriers, 'modelling' for opportunity and motivation barriers. Finally we will select specific behaviour change techniques e.g. education, goal setting, that focus on the specific functions we have identified. This will form the intervention programme theory that we draw

For peer review only - http://bmjopen.bmj.com/site/about/guidelines.xhtml

on for the next stage of the study, as documented through a logic diagram. Themes that do not map clearly onto the COM-B framework will also inform the programme theory e.g. contextual themes at the community and health system levels..

Stakeholder co-design workshops

Following evaluation of the focus groups and interviews our stakeholders, 12-15 patients, healthcare providers, commissioners and community leaders, will be invited to participate in a series of 2-3 half-day workshops, held in community locations. The workshops will seek to gain stakeholder involvement in developing the details of the interventions, determining the setting, the media channels, structure and delivery. In the first workshop the research team will feed back the findings of the focus groups and interviews; anonymised interview extracts will be presented to illustrate the key themes and issues that were identified. The stakeholders will be asked to discuss the themes and behavioural targets in small groups, using directed tasks/questions to facilitate the discussions. Following the small group discussions the researchers will facilitate discussion as a whole to clarify/confirm interpretation; open discussion/debate will be encouraged to examine the themes in depth and for all stakeholders to agree a mutual understanding.

In the second workshop elements of the proposed intervention will be presented for comment, refining and development. Using scenarios, the stakeholders will be asked to brainstorm, in small groups, key issues relating to the scenarios. For example, the moderator will present scenarios relating to the intervention setting and the attendees will be asked to discuss and identify the pros and cons of each, and then feed back their discussions to the other attendees. The research team will then facilitate cross-discussion between groups to develop the conclusions and a consensus.

For peer review only - http://bmjopen.bmj.com/site/about/guidelines.xhtml

In the final workshop draft intervention materials, developed from workshops 1 and 2, will be presented. For example, media channels that could be used to promote behaviour change such as testimonials, story-telling, and cooking demonstrations. The stakeholders will be divided into small groups to discuss and provide feedback on the acceptability of the components of the intervention and identify potential barriers to engagement. Following the small group discussions the researchers will facilitate feedback and encourage discussion as a whole to clarify/confirm the researcher's interpretation. The intervention template may be further refined, and will be developed into the detailed programme.

The workshop discussions will be digitally audio-recorded to allow the researchers to revisit the discussions as they develop the intervention details. The workshops will be analysed thematically at a descriptive level in order to inform the intervention design rather than for the development of conceptualisations. If the discussions are lengthy and complex they will be transcribed for this, if not, the researchers will take notes as they listen to the audio-recordings.

Phase 2 – Evaluation of HEAL-D; a culturally-tailored diet and lifestyle intervention for managing T2D in African and Caribbean communities

In phase 2 a feasibility study, with an embedded process evaluation, will be conducted to address the following objectives:

1. Estimate the effect of the intervention on a range of diabetes-related outcomes including HbA1c, weight, waist circumference, blood pressure, dietary intake, physical activity levels, diabetes knowledge, quality of life to inform an effectiveness trial.
2. Evaluate the acceptability of the intervention.
3. Evaluate implementation of the intervention, including fidelity and contextual factors.

For peer review only - http://bmjopen.bmj.com/site/about/guidelines.xhtml

4. Evaluate the feasibility of trial procedures.

Study Design

The feasibility study will use a randomised controlled design (Figure 1), with individual patients as the unit of randomisation, evaluating HEAL-D against usual care. In addition there will be a cohort of phase 1 co-design patients who will be allocated to the intervention arm (not randomised) because their involvement in the intervention design phase would contaminate the control arm. These patients are included in the feasibility study to allow us to evaluate the impact of former involvement on intervention engagement, acceptability and ownership. The RCT design has been chosen to [a] test the feasibility of recruiting and retaining a control arm, [b] define what constitutes 'usual care' and the variability within that, [c] determine the feasibility of using individual randomisation in a full-scale trial, looking particularly at issues of sample size and contamination between study arms, and [d] evaluate the impact of intervention development involvement on subsequent uptake and acceptability.

Participants

Participants will principally be recruited from General Practice in the London Boroughs of Lambeth and Southwark through screening of referrals for structured education and letters of invitation to patients with established T2D. In addition participants from the phase 1 co-design study will be invited to participate, and self-referral methods will also be used, for example posters and advertisements in community locations.

Patients with diagnosed T2D who are of African or Caribbean ethnicity and with capacity to provide fully informed consent to participation in research will be eligible to participate in the trial. Ethnicity will be self-declared using the standard NHS ethnicity categorisation questionnaire. Patients who are unable to communicate in English and patients with complex therapeutic dietary needs may be ineligible to participate if their individual needs are deemed

For peer review only - http://bmjopen.bmj.com/site/about/guidelines.xhtml

incompatible with the aims of the intervention. This is because the intervention will provide general diet and lifestyle advice for the self-management of T2D in a group setting; in cases of patients with certain comorbidities e.g. advanced renal disease, the intervention may be inappropriate for the individual, and the group nature of the intervention will prevent their individual needs from being addressed.

A pragmatic sample size of 120 patients is anticipated to be sufficient to evaluate the programme, allowing for 20% drop-out/non-completion; 80 patients will be randomised, 40 in each arm, and a further cohort of patients (n=40) from phase 1 will be allocated to the intervention arm without randomisation. As this is a feasibility trial it is not powered to detect statistically significant intervention effects. A primary objective of the study is to provide estimates of key parameters such as potential effect sizes, recruitment and retention rates of the trial and participation rates of the programme, so that the optimal design of a full-scale trial can be determined.

Intervention and control arms

Participants in the control arm will continue with usual care deemed appropriate and delivered by their primary care team, which may include referral to group structured education and/or one-to-one consultations with healthcare professionals.

Participants in the intervention arm will be offered the HEAL-D programme, which will deliver a curriculum of culturally-tailored, evidence-based diet and physical activity education and behavior change in a group setting. The details of each session, particularly the behavior change techniques and corresponding activities will be identified through the phase 1 co-design work.

The proposed curriculum is as follows:

1. *'Diabetes: it's in your hands'*: an introduction to self-management principles.

For peer review only

For peer review only - http://bmjopen.bmj.com/site/about/guidelines.xhtml

2. *'Get moving!'*: the role of physical activity in type 2 diabetes management.
3. *'Taking control'*: understanding carbohydrates & portion sizes.
4. *'Shape up!'*: weight management for diabetes.
5. *'Drop the pressure'*: managing cardiovascular health.
6. *'Plans for life: yes you can!'*: long-term maintenance of healthy lifestyles.

The sessions will consist of group-based education and behavior change techniques/activities, and participation in instructor-led physical activity.

The education sessions will be delivered through educator-led interactive discussion, however support materials will be provided to reinforce the learning e.g. videos and written information booklets detailing evidence-based diet and physical activity guidance, which is culturally tailored for the African and Caribbean communities.

Data Collection

Participants will attend a baseline and post-intervention follow-up assessment visit at 26-32 weeks. Data collection will focus on the following:

A. Estimating the effect of the intervention on potential trial outcomes
    - HbA1c, total- HDL- & LDL-cholesterol, triglycerides: a 5ml venous blood sample will be taken for analysis of HbA1c & lipids.
    - Body weight, height and body mass index (BMI): body weight will be measured using digital scales, with the patient wearing light clothing (without shoes), to the nearest 0.1 kg. Height will be measured, using a stadiometer, without shoes.
    - Waist circumference: measured using a flexible tape, with the patient wearing only light clothing, at the mid-point between the lowest rib and the iliac crest.
    - Systolic and diastolic blood pressure: the mean of three seated readings, taken using an automated sphygmomanometer.

For peer review only - http://bmjopen.bmj.com/site/about/guidelines.xhtml

- Diet & physical activity behaviours: dietary intake will be assessed through completion of a 24-hour diet recall, using the structured multiple pass interview method, and physical activity through 3-day Actiwatch accelerometer assessment and completion of the International Physical Activity Questionnaire (IPAQ).

B. Evaluating intervention mechanisms

The following validated self-complete questionnaires will be administered:

- Perceived Diabetes & Dietary Competence (PDDC) questionnaire
- Short Diabetes Knowledge Instrument (SDKI)
- Diabetes Empowerment Scale- Short Form (DEC-SF)
- Social support: Multidimensional Scale of Perceived Social Support (PSS)
- Quality of life: EQ-5D-3L & PAID-5

C. Evaluating acceptability & implementation of the intervention, and feasibility of trial procedures

Process evaluation is an essential part of testing complex interventions, allowing researchers to refine the theory by which the intervention brings about change, understand how the multiple components of the intervention may interact and how the intervention is implemented in the 'real world' setting (30). Our process evaluation will assess implementation and acceptability of the intervention. In terms of implementation, we will assess: 1) the reach of the intervention; 2) completion of the intervention (dose); 3) fidelity of the intervention, this includes coverage of core materials and learning objectives during delivery, and the extent to which the programme is delivered in accordance with the delivery manual; 4) quality of delivery; 5) barriers and facilitators to the uptake of the intervention in current care pathways.

For peer review only - http://bmjopen.bmj.com/site/about/guidelines.xhtml

375 Intervention acceptability will be explored by assessing participants' perceptions and experiences of the intervention, and how this varies across different settings and across the course of the programme. Acceptability of HEAL-D will be assessed through quantitative and qualitative data. Quantitative measures will include attendance records. Qualitative data will explore the experiences of participating in the sessions and the activities. Sustainability will be considered by assessing the scope for the intervention to be embedded within current care pathways, and contextual factors that may determine decision-making around continuance. Table 1 presents the research questions addressed by the process evaluation, and how they map onto the key domains being examined. The process evaluation data types and how they relate to the research questions is summarised in Table 2; details of our process evaluation data collection is provided in Table 3.

For peer review only

ETHICS & DISSEMINATION

This paper presents the protocol for the design and feasibility testing of HEAL-D, a culturally-tailored diet and physical activity intervention for the self-management of type 2 diabetes in UK African and Caribbean communities. In this work we will use rigorous complex intervention methodology to develop our intervention and evaluate its implementation prior to the design of a definitive trial of HEAL-D. The study protocol has been approved by the Fulham: London Research Ethics Committee (17-LO-1954); all participants will provide written consent prior to participation. All data will be anonymised and data protection protocols followed.

The study findings will be disseminated to the scientific community via conference presentations and peer-reviewed manuscripts, and to healthcare professionals via national and local clinical networks. The findings of the study will be communicated to our participants and local communities via the community networks and figureheads who we have engaged in our participatory methods; we will give presentations at church events and publish a newsletter via our study website (www.heal-d.co.uk).

For peer review only - http://bmjopen.bmj.com/site/about/guidelines.xhtml

403 **Abbreviations**

404 AfC African-Caribbean

405 COM-B Capability Opportunity Motivation Behaviour

406 HEAL-D Healthy Eating & Active Lifestyles for Diabetes

407 MRC Medical Research Council

408 NHS National Health Service

409 T2D Type 2 diabetes

410

411 **Declarations**

412 The study protocol has been approved by the Health Research Authority (London Fulham
413 Research Ethics Committee; 17/LO/1954); all participants will provide written consent prior
414 to participation.

415 **Consent for publication**

416 Not applicable.

417 **Availability of data and materials**

418 Not applicable.

419 **Competing interests**

420 The authors declare that they have no competing interests.

421 **Funding**

For peer review only - http://bmjopen.bmj.com/site/about/guidelines.xhtml

This report is independent research arising from a Career Development Fellowship (Louise M. Goff, CDF-2015-08-006) supported by the National Institute for Health Research. The views expressed in this publication are those of the author(s) and not necessarily those of the NHS, the National Institute for Health Research or the Department of Health.

Author contributions

All authors have made substantial contributions to this study. LMG, CR and SH were responsible for the conception and design of the study. LMG, CR, SH and AM developed the protocol and study approach. LMG drafted the manuscript. All authors read, revised and approved the final manuscript. LMG is guarantor.

Acknowledgements

Not applicable.

Figure legends

Figure 1. Overview of HEAL-D: development (phase 1) and feasibility evaluation (phase 2) of a culturally-tailored diet and lifestyle intervention for type 2 diabetes in African and Caribbean communities

Figure 2. Applying the COM-B behaviour change framework to the development of the HEAL-D intervention; identifying theory of change

Figure 3. Topic guides for patient focus groups & stakeholder interviews

For peer review only

Table 1. HEAL-D process evaluation domains and research questions

Process Evaluation Domain	Research Question
Mechanisms of Change	Are the intervention's mechanisms of change operationalised as hypothesised?
	How is the operationalisation of the mechanisms of change influenced by contextual factors?
	Does the interaction of the mechanisms of change with contextual factors give rise to unintended effects?
Programme Differentiation and Usual Practice	Is the HEAL-D intervention differentiable from 'usual practice'?
	Is there contamination of usual practice in control patients by receipt of the HEAL-D intervention?
Implementation	What is the reach of the HEAL-D intervention?
	How many patients complete the HEAL-D intervention?
	Are the HEAL-D components and sessions delivered with fidelity and what is the nature of any adaptions undertaken?
	Are there differences in the delivery of the HEAL-D sessions between sites, and what gives rise to any differences?
	How well are the HEAL-D components and sessions delivered?
	What are the barriers and facilitators to the implementation of the HEAL-D programme?
Acceptability	Is the HEAL-D intervention acceptable to commissioners, healthcare professionals, and patients?
Sustainability	How likely is the HEAL-D intervention to be sustainable and what factors might ensure sustainability?

For peer review only - http://bmjopen.bmj.com/site/about/guidelines.xhtml

Table 2. Mapping of research questions, process evaluation measures and data sources in HEAL-D feasibility study

Research Domain	Research Question	Data Source	Informant	Procedure of Data Collection	Time of Data Collection
Mechanisms of Change	Operationalisation of intervention mechanisms. Contextual factors. Unintended effects.	A. Patient questionnaires	Patients (n≈120)	Self-assessment; completion of paper questionnaire	Baseline & 26-32 week follow-up
		B. Observation of interventions sessions	HEAL-D educators (n≈4); HEAL-D patients	Independent assessment of intervention by research team (n=2); completion of observation schedules	During programme delivery
		C. HEAL-D course fidelity checklist and record of activities & materials	HEAL-D educators (n≈4); HEAL-D attendees	Self-assessment; completion of checklists and materials logs	During programme delivery
		D. HEAL-D evaluation forms	HEAL-D patients	Self-assessment; completion of evaluation forms	During programme delivery
		E. HEAL-D educator interview	HEAL-D educators	Interview led by study team	At the end of each delivery
		F. Patient interview	Patients	Interview led by study team	During & at the end of programme delivery
		G. Patient focus group	HEAL-D patients	Focus group led by study team	At the end of programme delivery
Programme Differentiation and Usual Practice	Differentiation.	A. Patient questionnaires	Patients (n≈120)	Self-assessment; completion of paper questionnaire	Baseline & 26-32 week follow-up
	Contamination.	F. Patient interview	Patients	Interview led by study team	26-32 wks follow-up
Implementation	Reach.	H. Attendance records	Patients	Participant completion; programme registers	During programme

For peer review only

For peer review only - http://bmjopen.bmj.com/site/about/guidelines.xhtml

	Completion. Fidelity. Quality. Barriers and facilitators.				delivery
		A. Patient questionnaires	Patients (n≈120)	Self-assessment; completion of paper questionnaire	Baseline & 26-32 week follow-up
		B. Observation of intervention sessions.	HEAL-D educators (n≈4); HEAL-D patients	Independent assessment of intervention by research team (n=2); completion of observation schedules	During programme delivery
		E. HEAL-D educator interview	HEAL-D educators	Interview led by study team	At the end of programme delivery
		I. Healthcare professional interview	Healthcare professionals	Interview led by study team	At the end of programme delivery
Acceptability	Acceptability.	J. Commissioner interview	Commissioners	Interview led by study team	At the end of programme delivery
		G. Patient focus group	HEAL-D patients	Focus group led by study team	At the end of programme delivery
		I. Healthcare professional interview	Healthcare professionals	Interview led by study team	At the end of programme delivery
		E. HEAL-D educator interview	HEAL-D educators	Interview led by study team	At the end of programme delivery
Sustainability	Sustainability.	J. Commissioner interview	Commissioners	Interview led by study team	At the end of programme delivery
		G. Patient focus group	HEAL-D patients	Focus group led by study team	At the end of programme delivery
		I. Healthcare professional interview	Healthcare professionals	Interview led by study team	At the end of programme
		E. HEAL-D educator			

For peer review only

	interview	HEAL-D educators	Interview led by study team	delivery<br>At the end of programme delivery

For peer review only

For peer review only - http://bmjopen.bmj.com/site/about/guidelines.xhtml

Table 3. Details of process evaluation data collection methods

A. Patient questionnaires

Questionnaire data will test the proposed theory of change underpinning the intervention through measurement of knowledge, motivation, and social support.

B. Observation of intervention sessions

The research team will observe the HEAL-D sessions to quantitatively assess: coverage of curriculum; use of supporting materials and behaviour change techniques; quality of delivery; and participant engagement (binary score or a five-point Likert scale). Observers will qualitatively document any information relevant for understanding the quantitative assessment, and other issues of importance e.g. course adaptations and general contextual observations. Observations will inform the focus group and interview topic guides for educators, patients and healthcare professionals.

C. HEAL-D course fidelity checklist and record of activities & materials

Educators will list any resources, activities, examples or discussions that were additional to the standardised schedule. These data will be used to assess fidelity, and compare intervention deliveries and contextual impacts.

D. HEAL-D evaluation forms

Evaluation forms will use five-point Likert scales to record self-reported increase in knowledge, motivation, social support, and views on quality of course content, structure, format and delivery.

E. HEAL-D educator interviews

Semi-structured interviews will be conducted with the intervention educators to explore: mechanisms underpinning the behaviour change processes; preparedness to deliver the intervention components; experiences of delivery, including barriers and facilitators; fidelity and motivations for any adaptations undertaken; and perceived acceptability.

F. Patient interviews

Semi-structured interviews will be conducted with patients from both arms of the study, purposively sampled to ensure representation from all deliveries of the intervention and a spread of gender, age, ethnicity and employment status. Data will explore: experiences of intervention and control; perceived acceptability of sessions and session components; reasons for attendance and non-attendance, including contextual factors; impact of sessions on behaviour, including any behaviour change that has occurred. Among control patients issues of contamination will be explored and perceptions of 'usual care' will be discussed.

G. Patient focus groups

Focus groups will be conducted with patients from the intervention arm to explore views of the intervention, including: acceptability; effectiveness at promoting behaviour change, and perceived barriers and facilitators to this.

H. Attendance records

Attendance records will assess reach (numbers completing the intervention), and retention in the programme and patterns in these according to gender, age, ethnicity and employment status.

I. Healthcare professional interviews

Semi-structured interviews will be conducted with healthcare professionals involved in the

For peer review only - http://bmjopen.bmj.com/site/about/guidelines.xhtml

primary care management of diabetes to assess intervention awareness, acceptability and effectiveness.

J. Commissioner interviews

Semi-structured interviews will be conducted with commissioner representatives to assess intervention acceptability, fit with organisational priorities, and the feasibility of sustained resource allocation to the HEAL-D intervention if found to be successful.

For peer review only

For peer review only - http://bmjopen.bmj.com/site/about/guidelines.xhtml

REFERENCES

1. Estimating the current and future costs of Type 1 and Type 2 diabetes in the United Kingdom, including direct health costs and indirect societal and productivity costs [Internet]. York Health Economics Consortium Ltd. 2012.
2. Wilson C, Alam R, Latif S, Knighting K, Williamson S, Beaver K. Patient access to healthcare services and optimisation of self-management for ethnic minority populations living with diabetes: a systematic review. Health & social care in the community. 2012;20(1):1-19.
3. Becker E, Boreham R, Chaudhury M, Craig R, Deverill C, Doyle M, et al. Health Survey for England 2004. The health of minority ethnic groups. London: Joint Health Surveys Unit, National Centre for Social Research, Department of Epidemiology and Public Health at the Royal Free and University College Medical School, 2006.
4. Ng M, Fleming T, Robinson M, Thomson B, Graetz N, Margono C, et al. Global, regional, and national prevalence of overweight and obesity in children and adults during 1980-2013: a systematic analysis for the Global Burden of Disease Study 2013. Lancet. 2014;384(9945):766-81.
5. Lanting LC, Joung IM, Mackenbach JP, Lamberts SW, Bootsma AH. Ethnic differences in mortality, end-stage complications, and quality of care among diabetic patients: a review. Diabetes Care. 2005;28(9):2280-8.
6. Healthcare for London. Diabetes guide for London. 2009.
7. London Assembly. Review of diabetes care in London. 2013.
8. Department of Health. Improving quality of life for people with long term conditions. London, UK: Department of Health, 2013.
9. Department of Health. National service framework for diabetes standards. 2001.
10. National Institute for Health and Care Excellence. Type 2 diabetes in adults: management (NG28). 2015.
11. Lakhani M. No patient left behind: how can we ensure world class primary care for black and minority ethnic people? London: Department of Health; 2008.
12. Healthcare Commission. Managing diabetes: improving services for people with diabetes. 2007.
13. Brown K, Avis M, Hubbard M. Health beliefs of African-Caribbean people with type 2 diabetes: a qualitative study. The British journal of general practice : the journal of the Royal College of General Practitioners. 2007;57(539):461-9.
14. Sarkar U, Piette JD, Gonzales R, Lessler D, Chew LD, Reilly B, et al. Preferences for self-management support: findings from a survey of diabetes patients in safety-net health systems. Patient education and counseling. 2008;70(1):102-10.
15. Stone MA. Making education in diabetes culturally appropriate for patients. Practice Nursing. 2006;17(12):5.
16. Cross T, Bazron B, Dennis K, Isaacs M. Towards a culturally competent system of care. Washington D.C.: Georgetown University Child Development Center, CASSP Technical Assistance Center; 1989.
17. Hawthorne K, Robles Y, Cannings-John R, Edwards AG. Culturally appropriate health education for type 2 diabetes mellitus in ethnic minority groups. The Cochrane database of systematic reviews. 2008(3):CD006424.
18. Liu JJ, Davidson E, Bhopal RS, White M, Johnson MRD, Netto G, et al. Adapting health promotion interventions to meet the needs of ethnic minority groups: mixed-methods evidence synthesis. Health Technol Assess. 2012;16(44).
19. Attridge M, Creamer J, Ramsden M, Cannings-John R, Hawthorne K. Culturally appropriate health education for people in ethnic minority groups with type 2 diabetes mellitus. Cochrane Database of Systematic Reviews. 2014(9):CD006424.
20. Medical Research Council. Developing and evaluating complex interventions: new guidance. 2006.

For peer review only - http://bmjopen.bmj.com/site/about/guidelines.xhtml

21. Dyson PA, Kelly T, Deakin T, Duncan A, Frost G, Harrison Z, et al. Diabetes UK evidence-based nutrition guidelines for the prevention and management of diabetes. Diabetic medicine : a journal of the British Diabetic Association. 2011;28(11):1282-8.

22. O'Mara-Eves A, Brunton G, McDaid D, Oliver S, Kavanagh J, Jamal F, et al. Community engagement to reduce inequalities in health: a systematic review, meta-analysis and economic analysis. Public Health Res. 2013;1(4).

23. Norris SL, Nichols PJ, Caspersen CJ, Glasgow RE, Engelgau MM, Jack L, et al. Increasing diabetes self-management education in community settings. A systematic review. American journal of preventive medicine. 2002;22(4 Suppl):39-66.

24. Di Noia J, Furst G, Park K, Byrd-Bredbenner C. Designing culturally sensitive dietary interventions for African Americans: review and recommendations. Nutrition reviews. 2013;71(4):224-38.

25. Sumlin LL, Garcia AA. Effects of food-related interventions for African American women with type 2 diabetes. The Diabetes educator. 2012;38(2):236-49.

26. Samuel-Hodge CD, DeVellis RF, Ammerman A, Keyserling TC, Elasy TA. Reliability and validity of a measure of perceived diabetes and dietary competence in African American women with type 2 diabetes. The Diabetes educator. 2002;28(6):979-88.

27. Abubakari AR, Jones MC, Lauder W, Kirk A, Anderson J, Devendra D, et al. Ethnic differences and socio-demographic predictors of illness perceptions, self-management, and metabolic control of type 2 diabetes. International journal of general medicine. 2013;6:617-28.

28. National Institute for Health and Care Excellence. Behaviour change: individual approaches. 2014 Public health guidelines (PH49).

29. Michie S. Designing and implementing behaviour change interventions to improve population health. Journal of health services research & policy. 2008;13 Suppl 3:64-9.

30. Moore GF, Audrey S, Barker M, Bond L, Bonell C, Hardeman W, et al. Process evaluation of complex interventions: Medical Research Council guidance. BMJ. 2015;350:h1258.

For peer review only

For peer review only - http://bmjopen.bmj.com/site/about/guidelines.xhtml

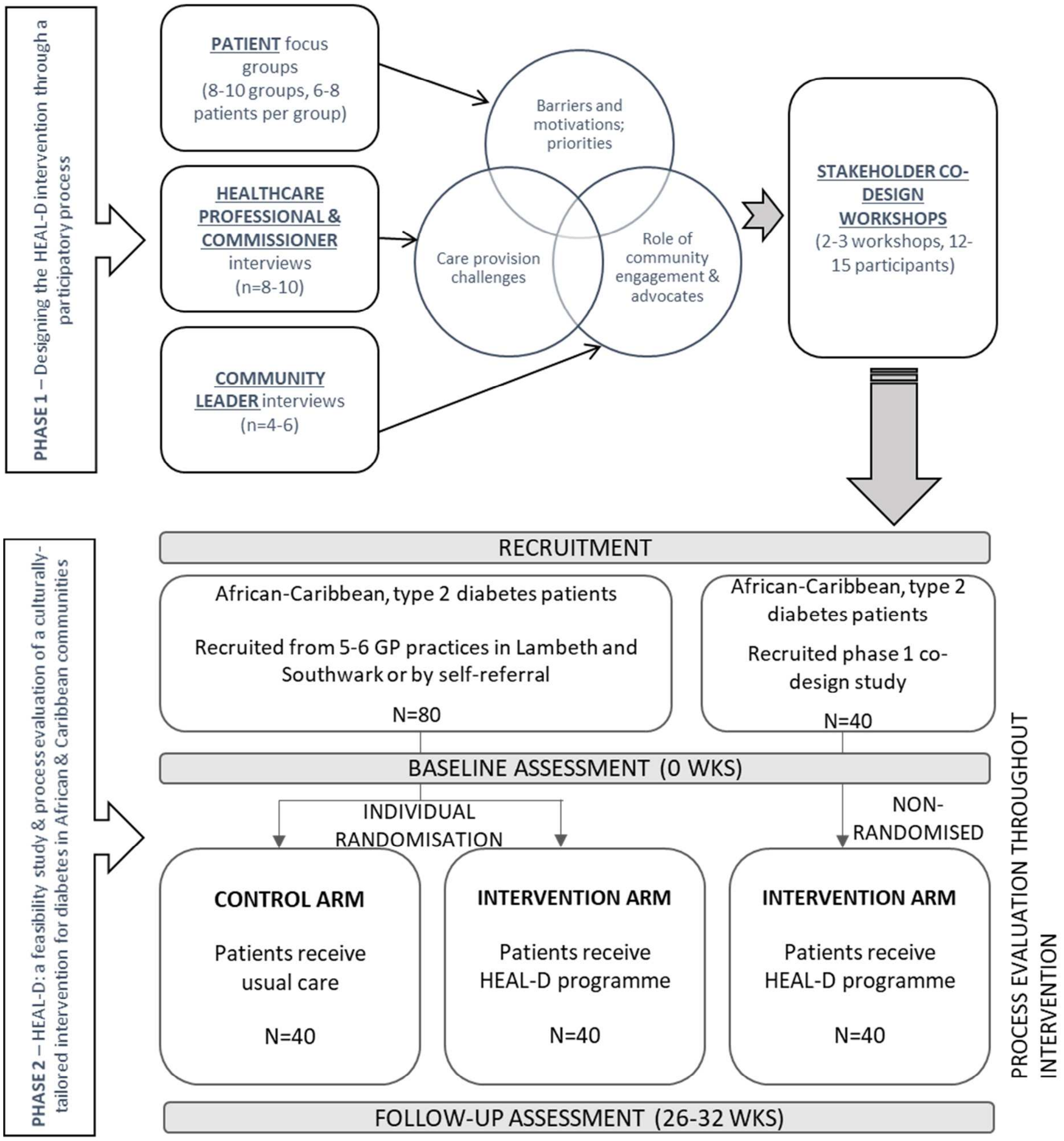
PHASE 1 – Designing the HEAL-D intervention through a participatory process
PATIENT focus groups (8-10 groups, 6-8 patients per group)
HEALTHCARE PROFESSIONAL & COMMISSIONER interviews (n=8-10)
COMMUNITY LEADER interviews (n=4-6)
Barriers and motivations; priorities
Care provision challenges
Role of community engagement & advocates
STAKEHOLDER CO-DESIGN WORKSHOPS (2-3 workshops, 12-15 participants)
PHASE 2 – HEAL-D: a feasibility study & process evaluation of a culturally-tailored intervention for diabetes in African & Caribbean communities
RECRUITMENT
African-Caribbean, type 2 diabetes patients
Recruited from 5-6 GP practices in Lambeth and Southwark or by self-referral
N=80
African-Caribbean, type 2 diabetes patients
Recruited phase 1 co-design study
N=40
BASELINE ASSESSMENT (0 WKS)
INDIVIDUAL RANDOMISATION
NON-RANDOMISED
CONTROL ARM
Patients receive usual care
N=40
INTERVENTION ARM
Patients receive HEAL-D programme
N=40
INTERVENTION ARM
Patients receive HEAL-D programme
N=40
PROCESS EVALUATION THROUGHOUT INTERVENTION
FOLLOW-UP ASSESSMENT (26-32 WKS)

Behaviour goal: Walk 10,000 steps per day at moderate intensity	
**Capability**	Knowledge: Does the target group know:<br>- Why walking 10,000 steps would help diabetes management?<br>- What moderate intensity is and how to measure it?<br><br>Behavioural regulation: Does the target group know how to:<br>- Plan to fit the activity in to their daily life?<br>- Remember to do the activity?<br>- Prioritise this activity over others?<br>- Record & measure and self-monitor their activity?<br><br>Physical skills: Does the target group:<br>- Have the physical stamina to walk 10,000 steps at this intensity?
**Opportunity**	Environmental context & resources:<br>- Is there somewhere safe to walk in the neighbourhood?<br>- Do the patients have appropriate equipment e.g. suitable trainers or walking shoes?<br>- Can they afford a pedometer or some means of measuring their steps?<br><br>Social influences (what interpersonal influences cause individuals to change their thoughts, feelings or behaviours?)<br>- It is culturally acceptable to walk for exercise?<br>- What is the social norm amongst immediate friends and family?<br>- What positive or negative views do others have that may influence activity?<br>- Are there any positive role models?<br>- Are there competing demands e.g. pressure to spend leisure time with family or at church?<br>- Would group support be motivating?
**Motivation**	Reflective (conscious) motivation:<br>- How optimistic do the patients feel they can achieve the goal?<br>- Do they intend to do the behaviour (stages of change model)?<br>- What emotions may help or hinder? e.g. do they fear injury?<br>- What other emotions may conflict? e.g tiredness, depression, stress<br><br>Automatic (innate drivers):<br>- What are established habit patterns?<br>- What are routines/thought/behaviours set up by previous experience?

For peer review only

Patient focus groups

- Knowledge and perceptions of diabetes, and diet and lifestyle advice for managing diabetes
- Current practices relating to diabetes self-care, and diet and lifestyle
- Health concerns/priorities in relation to diabetes
- Motivations and barriers/difficulties relating to diabetes self-care, weight management and diet and lifestyle
- Experiences and perceptions of diabetes care/education, and barriers to accessing care
- Experiences of behaviour change in relation to diabetes, weight, diet and lifestyle – successes and failures
- Role of family/friends/communities in influencing and shaping knowledge and behaviours in relation to diabetes, diet and lifestyle

Community leader interviews (including religious leaders)

- Health problems affecting the community and diabetes impact on health within this context
- Attitude of the community towards health, medicines, doctors
- Role of community leaders in promoting health and community activities
- Diabetes health promotion activities within the community. What worked and what didn't?
- Barriers and facilitators to positive diabetes behaviours within the community
- Advice about engaging the community: Who are the role models? What will engage and help people? How can healthcare & community work together?

Healthcare professional interviews

- Experience of supporting African & Caribbean patients – what happens? What are the issues? How could things be improved? What factors make successful T2D management likely?
- Patient beliefs and motivations
- Involvement in community activities and experience of working with community leaders and lay educators and suggestions to improve partnerships
- Difficulties & challenges with offering a tailored lifestyle intervention

For peer review only

BMJ Open

Healthy Eating and Active Lifestyles for Diabetes (HEAL-D): study protocol for the design and feasibility trial, with process evaluation, of a culturally-tailored diabetes self-management programme for African-Caribbean communities

Journal:	*BMJ Open*
Manuscript ID	bmjopen-2018-023733.R1
Article Type:	Protocol
Date Submitted by the Author:	05-Nov-2018
Complete List of Authors:	Goff, Louise; Kings College Lonodn, Diabetes and Nutritional Sciences<br>Moore, Amanda; Kings College Lonodn, Diabetes and Nutritional Sciences<br>Rivas, Carol; University of Southamptom, Faculty of Health Sciences<br>Harding, Seeromanie; King\'s College London Division of Diabetes and Nutritional Sciences, Diabetes & Nutritional Sciences Division
**Primary Subject Heading**:	Diabetes and endocrinology
Secondary Subject Heading:	Nutrition and metabolism
Keywords:	type 2 diabetes, ethnicity, culture, diet, lifestyle, education

SCHOLARONE™
Manuscripts

Healthy Eating and Active Lifestyles for Diabetes (HEAL-D): study protocol for the design and feasibility trial, with process evaluation, of a culturally-tailored diabetes self-management programme for African-Caribbean communities

Louise M. Goff[1#], Amanda P. Moore[1], Carol Rivas[2], Seeromanie Harding[1]

[1]King's College London, Departments of Diabetes & Nutritional Sciences, School of Life Course Sciences, Faculty of Life Sciences & Medicine, [2]Institute of Education, University College London

[#]Corresponding author: Dr Louise M. Goff, Diabetes Research Group, King's College London, Franklin-Wilkins Building, Room 3.87, Waterloo Campus, King's College London, London SE1 9NH. T: 020 7848 4273; E: louise.goff@kcl.ac.uk

For peer review only

For peer review only - http://bmjopen.bmj.com/site/about/guidelines.xhtml

ABSTRACT

Introduction

Black-British communities are disproportionately burdened by type 2 diabetes (T2D) and its complications. Tackling these inequalities is a priority for both healthcare providers and patients. Culturally-tailored diabetes education provides long-term benefits superior to standard care but to date such programmes have only been developed in the USA. The Healthy Eating and Active Lifestyles for Diabetes (HEAL-D) programme of research aims to develop a culturally-tailored T2D self-management programme for Black-British communities; and to evaluate its delivery, acceptability and the feasibility of conducting a future effectiveness trial of HEAL-D.

Methods & analysis

Informed by Medical Research Council (MRC) Complex Interventions guidance this research will rigorously develop and evaluate the implementation of the HEAL-D intervention to understand the feasibility of conducting a full-scale effectiveness trial. In Phase 1 the intervention will be developed. The intervention curriculum will be based on existing evidence-based guidelines for diet and lifestyle management of T2D; co-design methods will be used to foster community engagement, identify the intervention's underpinning theory; identify the optimal structure, format and methods of delivery, ascertain adaptations that are needed to ensure cultural sensitivity of the content, and understand issues of implementation. In Phase 2 process evaluation methods will evaluate the delivery and acceptability of HEAL-D; the feasibility of conducting a future effectiveness trial will also be evaluated, particularly feasibility of randomisation, recruitment, retention, and contamination, and identify potential primary outcomes for a future trial.

Ethics & dissemination

For peer review only

For peer review only - http://bmjopen.bmj.com/site/about/guidelines.xhtml

This study is funded by a National Institute of Health Research Fellowship (CDF-2015-08-006). It has been approved by the Fulham: London Research Ethics Committee (17-LO-1954). Results will be disseminated at national and international conferences, in peer-reviewed publications and through local and national clinical diabetes networks.

Trial Registration: this trial is registered with www.clinicaltrials.gov, identifier: NCT03531177.

STRENGTHS AND LIMITATIONS OF THIS STUDY

- This study will employ rigorous complex intervention methodology to develop and evaluate the implementation of a culturally-tailored diabetes self-management intervention.
- Our intervention, HEAL-D, will be designed using a 'bottom-up' approach, employing participatory co-design methods to foster community engagement and partnership.
- We will identify the cultural adaptations of our intervention and its underpinning theoretical basis through thematic analysis and the COM-B behavior change framework.
- The feasibility study will provide us with key information about the feasibility of running a full-scale trial of HEAL-D.
- Process evaluation methods will enable us to understand how and why the intervention is effective or ineffective.

Keywords: African, Caribbean, ethnicity, type 2 diabetes, education, self-management, diet, lifestyle

For peer review only - http://bmjopen.bmj.com/site/about/guidelines.xhtml

INTRODUCTION

Type 2 diabetes (T2D) affects approximately 3 million people in England and consumes around 10% of the National Health Service (NHS) budget, estimated at almost £9 billion in 2011 and predicted to rise to 17% of the NHS budget by 2035 (1). Diabetes and its associated complications place an illness burden on patients and carers, which disproportionately affects those from ethnic minority backgrounds (2). The estimated prevalence of T2D is up to 3 times higher for Black-British communities compared to White Europeans (3). T2D occurs, on average, 10 years earlier in Black-British people, the mean age of diagnosis is 48 years and approximately 25% of patients are under the age of 40 years (4). Furthermore, glycaemic control is worse at the time of diagnosis, requires greater medical management, and poorer outcomes are evident (5-7). The reasons for these disparities are not fully understood; while biological factors are involved, it is understood that a range of behavioural, lifestyle and health system factors play a role. Tackling these inequalities is a healthcare priority (8, 9).

Individuals of Black-British ethnicity form the second largest ethnic minority population in the United Kingdom (UK); around 4% of the population self-identify from this ethnic background (10). Around half of individuals are of Black African ancestry and a third of Black Caribbean ancestry (10). Growth in the Black-British communities is relatively recent, beginning mainly in response post-second world war appeals to citizens of the Commonwealth regions to assist with gaps in its labour market. This prompted a large influx of migrants in the 1950s from the Caribbean islands, particularly Jamaica. Migration from the African continent has been more recent, peaking around the 1980s; migrants from African nations currently form the largest growing ethnic minority group in the UK population (11). In some regions, such as London, Black-British communities may represent 30-40% of the local population and are therefore a 'majority-minority' community. Other demographic patterns are recognised; the age distribution of the Black African and Black Caribbean communities differs, with a larger

For peer review only - http://bmjopen.bmj.com/site/about/guidelines.xhtml

proportion of Black Caribbeans being aged 65 years and over, while in the Black African population a greater proportion are children and young adults. High rates of unemployment are evident, averaging around 12% compared to 4% in the White British population (11).

Poor access to diabetes healthcare is a significant issue for minority ethnic groups (2). In the UK the NHS provides care to all UK residents that is free at the point of delivery. First-line diabetes management is situated in primary care and aims to promote patient involvement and self-management (12), enabling patients to adopt a healthy lifestyle and to manage their diabetes through support and education (13). To achieve this, UK T2D management guidelines recommend that all patients attend a structured education course to teach them the principals of T2D self-management and that this be offered annually from the time of diagnosis (14). Courses are recommended to use a group structure; typically they use face-to-face delivery by a diabetes specialist nurse or dietitian, with lay educator co-delivery in some cases (14). Referral to such courses is audited and incentivised through the Quality Outcomes Framework (15). Ethnic minority groups report finding it more difficult to access primary care services (16) and are more likely to report that they have not had the opportunity to attend a diabetes education course than White populations (17). Specifically, African-Caribbean (AfC) communities often report a distrust of medical advice and a desire for natural, non-pharmacological therapies (18). Furthermore, healthcare professionals are perceived as lacking cultural understanding (19) and their advice as lacking cultural relevance (20) or being poorly adapted to culture and needs (18) despite their intentions; these issues may contribute to the poorer diabetes outcomes and increased morbidity experienced by AfC patients.

Culturally tailored healthcare is proposed to be one of the main ways in which healthcare disparities can be addressed (21-23) and is identified as a priority by patients (8). Culturally-tailored diabetes education has demonstrated greater improvements in diabetes control and knowledge than usual care, and the benefits are maintained long-term (22, 24). Culture is a

For peer review only - http://bmjopen.bmj.com/site/about/guidelines.xhtml

concept that is notoriously difficult to define but generally within healthcare it is thought of as ‘a set of attitudes, values, beliefs and behaviours shared by a group of people, communicated from one generation to the next’ (25). In their model for understanding cultural sensitivity in healthcare, Resnicow *et al.* (1999) described two dimensions in culture: surface and deep structures. Tailoring interventions to surface structures involves matching materials and messages to observable, "superficial" characteristics of a target population e.g. language and food, familiar to, and preferred by, the target audience. Deep structure involves incorporating the cultural, social, historical, environmental and psychological forces that influence the target health behaviours in the proposed target population. Whereas surface structure generally increases the "receptivity" or "acceptance" of messages, deep structure conveys salience (26). Culture is ever evolving for any group and it is important to recognise the diversity that exists within any one ‘cultural group’, which is particularly evident in migrant populations where second/third generations may have undergone significant acculturation. To date, culturally tailored interventions for the African diaspora have largely been based in the USA, and may not translate to UK healthcare structures or UK AfC communities whose cultural needs may be different (23).

Healthy Eating & Active Lifestyles for Diabetes (HEAL-D) is a two-phase programme of research focusing on the co-design of a culturally-tailored, evidence-based self-management programme for T2D in African and Caribbean communities, followed by a feasibility trial. The intervention curriculum will be based on existing evidence-based guidelines for T2D (14, 27) to enable it to have potential to be embedded into clinical practice; co-design methods will be used to identify the optimal structure, format and methods of delivery and to ascertain appropriate adaptations that are needed to ensure cultural sensitivity of the content. The purpose of this article is to present the protocol for HEAL-D.

For peer review only

PURPOSE & AIMS

The overall aims of this research are to develop a culturally-tailored, evidence-based self-management programme for managing T2D among AfC communities in primary care, called HEAL-D, and to determine the feasibility of evaluating HEAL-D through a future effectiveness trial.

The objectives are to:

1. Develop a self-management programme, based on existing evidence-based diet and lifestyle guidelines, appropriately tailored for AfC patients through co-design methods.
2. Establish the feasibility of conducting an effectiveness trial of HEAL-D, considering issues such as participation rates and potential effect sizes.

For peer review only

For peer review only - http://bmjopen.bmj.com/site/about/guidelines.xhtml

METHODS AND ANALYSIS

Guided by the Medical Research Council's Complex Interventions framework (28) (Figure 1), HEAL-D will consist of two distinct phases: phase 1 is a formative phase in which the intervention will be developed; and in phase 2, the intervention will be evaluated in a feasibility trial. Study recruitment will begin in April 2018; the study duration will be 36 months.

Phase 1 – Development of a culturally-tailored T2D self-management progamme

The process for the development of HEAL-D is outlined in Figure 2. Firstly, to ensure its potential to be embedded into future clinical practice, the HEAL-D curriculum will align with existing management recommendations and guidelines (14, 27):

Guidelines for diet and lifestyle management of T2D (27):

1. Achieve 5-10% weight loss or weight maintenance in those of healthy weight.
2. Undertake 150 minutes/week of moderate-to-vigorous intensity aerobic physical activity plus 2 sessions/week of strength training.
3. Balance carbohydrate intakes through portion control and promotion of low glycaemic index and wholegrain sources.
4. Limit saturated fat intake (<10% of energy intake), replace with mono-unsaturated fats.
5. Limit salt intake (<6g per day).
6. Consume oily fish at least twice per week.

Guidelines for T2D self-management education (14):

1. Offer structured education to adults with T2D and/or their family members or carers.
2. Offer group education programmes as the preferred option.

For peer review only - http://bmjopen.bmj.com/site/about/guidelines.xhtml

2. Ensure the education programme is theory-driven, evidence-based and meets the cultural, linguistic, cognitive and literacy needs of the population.

Drawing on the existing evidence base

Secondly it will draw on key themes reported in published literature relating to methodologies for adapting health promotion interventions for ethnic minority groups. These have been evaluated in a number of recent systematic reviews; aside from acknowledging the lack of UK-based studies, these reviews have made the following recommendations:

- Acknowledge the powerful influence of social networks on health beliefs and behaviours (29). Focus on community-level interventions; delivering care in a social context has been shown to promote engagement and be more effective than traditional individual-centred behavioural approaches (23).
- Foster community engagement to overcome issues of deep-rooted, historical distrust of medical advice and settings, develop and nurture trust between the researchers and community, and nurture the strong sense of collectivism and kinship networks that are evident amongst AfC communities.
- Employ participatory methods (e.g. patient involvement in intervention design, lay-led delivery of interventions), which are highly effective at improving health behaviours and self-efficacy across a number of conditions (30).
- Use community gathering places (e.g. faith institutions), which offer the benefit of cultural relevancy and may reach populations who would not normally access self-management education (31).

Identifying the intervention's theoretical basis

Behavioural interventions should have a theoretical under-pinning (28, 32) so that the changes that are expected, and how these will be achieved, can be predicted from consideration of

For peer review only - http://bmjopen.bmj.com/site/about/guidelines.xhtml

known behaviour change techniques. While there have been a number of interventions tailored to support diet and lifestyle behaviour change in AfC communities, their theoretical underpinning has rarely been drawn out or clearly presented. The theoretical underpinning of HEAL-D will be developed through a combination of key themes from the published literature and new primary research.

In the literature collectivism and the importance of social interaction for people of AfC ancestry is well reported (29), and the provision of a social support group, or inclusion of a family member, has been shown to be particularly effective in lifestyle interventions in African-American communities (33, 34). In USA interventions, researchers have proposed that a focus on facilitating/nurturing social support may be a particularly 'therapeutic and cost-effective public health strategy' for AfC communities (33). Notably, the majority of literature that identifies the drivers of health behaviours in AfC communities and may, therefore, inform the theoretical basis of an intervention, comes from the USA and it is not known to what extent these findings apply to AfC in other regions. One of the reasons we will use co-design methods will be to understand the relevance of these existing themes to the UK context and enable us to identify themes that are important to Black-British communities.

Co-designing the intervention through participatory methods

HEAL-D will use participatory co-design methods to engage patients, healthcare providers and community leaders (e.g. church leaders, community group leads) in focus groups, interviews and workshops in order to achieve the following:

1. Foster community engagement.
2. Identify the theoretical under-pinning of HEAL-D.
3. Identify appropriate cultural adaptations for the intervention.
4. Understand issues of intervention implementation.

For peer review only - http://bmjopen.bmj.com/site/about/guidelines.xhtml

Focus groups and interviews

Focus groups, 8-10 groups of 6-8 participants, will be conducted with patients with T2D of AfC ethnicity, recruited through local churches, mosques and community groups, as well as through GP practices in London. The focus groups will be conducted in local accessible community venues e.g. church hall, library, community centre. Patients will be purposively sampled to get a spread of socio-economic position, generational status and ancestral origins, as principal factors impacting on health status, healthcare access and cultural behaviours in these groups (35-37). Separate focus groups will be conducted with men and women, and patients of direct African *versus* Caribbean ancestry, as they report different cultural barriers/facilitators to lifestyle change (35, 36). A topic guide (Table 1) based on themes identified in the literature, will be used to steer discussions and ensure coverage of key themes whilst encouraging free discussion of opinion/perspective. Focus groups have been selected to enable us to understand normative needs, as suited to the development of a community intervention.

Semi-structured interviews will be conducted with 8-10 healthcare providers, including general practitioners, practice nurses, diabetes specialist nurses, diabetes specialist dietitians and commissioners. The interviews will cover issues relating to healthcare needs and engagement of AfC patients, experiences of delivering healthcare to AfC patients, and barriers and facilitators to working in partnership with community groups to deliver care for AfC communities (Table 1). Interviews have been selected for this part of the study to enable us to gather a full range of experiences and therefore optimise implementation.

Community leaders representing faith and non-faith institutions (n=4-6) will be invited to participate in semi-structured interviews. Leaders will be identified initially through existing networks e.g. Diabetes UK Community Champions initiative. Word-of-mouth and 'snow-

For peer review only

For peer review only - http://bmjopen.bmj.com/site/about/guidelines.xhtml

balling' techniques that are highly effective within these communities, will be used to recruit a wider network. The interviews will cover issues relating to the role of community networks in promoting health of AfC communities, sustaining health amongst community members, and opportunities for greater impact (Table 1).

Analysis

The focus groups and interviews will be digitally recorded and transcribed verbatim. The data will be analysed using the framework approach in NVivo (QSR International), theoretically driven by socio-ecological theory to identify themes relating to issues at the individual, family, community and healthcare delivery levels and how these influence self-efficacy and behaviour change. Our analysis will identify priority behaviours of focus for the intervention, key barriers and facilitators to behaviour change and healthcare engagement, favoured settings, and a rudimentary draft of the cultural adaptations. Deviant case analysis, that is consideration of cases that do not fit the general picture, will be undertaken, though our primary interest is in the commonalities as this is a community level intervention. Primary coding and development of a coding scheme will be carried out by a single researcher; a second researcher will independently use this coding scheme to code 20% of the data for cross-comparison, to improve dependability. This will provide methodological rigour required for confidence in the analysis of the qualitative data. The themes will be fed-back and discussed with a Service User Group (SUG), which will consist of representatives of patients, healthcare providers, and community leaders. The SUG will be set up to inform and guide each stage of the research plan and will be a forum through which the research team can seek the opinion of key stakeholders, in this case particularly relating to interpretation of the qualitative data and to ensure trustworthiness of conceptualisations. The SUG will also review research documents, such as patient information sheets and questionnaires, and provide feedback on their content and suitability for the communities of focus.

For peer review only - http://bmjopen.bmj.com/site/about/guidelines.xhtml

We will divide our data into behavioural 'barriers' and 'facilitators' where possible. To ascertain appropriate behaviour change techniques for our intervention (32) we will map our analysis onto the Capability-Opportunity-Motivation-Behaviour (COM-B) framework from the Behaviour Change Wheel (38) (Figure 3), and thence in each case consider the outcome behaviours that our intervention will aim to achieve, a worked example is shown in Figure 4. We will use the COM-B framework to identify appropriate *functions* of our intervention to optimise facilitators and overcome barriers to achievement of planned outcomes, e.g. 'education' for capability barriers, 'modelling' for opportunity and motivation barriers. Finally we will select specific behaviour change techniques e.g. education, goal setting, that focus on the specific functions we have identified. This will form the intervention theory that we will draw on for the next stage of the study, as documented through a logic diagram. We will also look to identify other themes that arise from the data, which might not map clearly onto the COM-B framework (e.g. contextual themes relating to the health system) but which may inform our intervention theory as well as help us to understand issues of implementation (e.g. favoured settings and timings).

Stakeholder co-design workshops

Following evaluation of the focus groups and interviews our stakeholders, 12-15 patients, healthcare providers, commissioners and community leaders, will be invited to participate in a series of 2-3 half-day workshops, held in community locations. The workshops will seek to gain stakeholder involvement in developing the details of the interventions. This will include determining the setting, the media channels, structure and delivery, as well as steering the research team to understand and respond to literacy and numeracy needs. The workshops will endeavour to reach a consensus opinion from attendees but where stakeholders have different needs and a consensus cannot be reached the research team will consult with the SUG to make decisions on the way forward and consider where there is scope for the intervention to be

For peer review only

For peer review only - http://bmjopen.bmj.com/site/about/guidelines.xhtml

structured to meet these different needs e.g. delivery in a range of settings. In the first workshop the research team will feed back the findings of the focus groups and interviews; anonymised interview extracts will be presented to illustrate the key themes and issues that were identified. The stakeholders will be asked to discuss the themes and behavioural targets in small groups, using directed tasks/questions to facilitate the discussions. Following the small group discussions the researchers will facilitate discussion as a whole to clarify/confirm interpretation; open discussion/debate will be encouraged to examine the themes in depth and for all stakeholders to agree a mutual understanding.

In the second workshop elements of the proposed intervention will be presented for comment, refining and development. Using scenarios, the stakeholders will be asked to brainstorm, in small groups, key issues relating to the scenarios. For example, the moderator will present scenarios relating to the intervention setting and the attendees will be asked to discuss and identify the pros and cons of each, and then feed back their discussions to the other attendees. The attendees will be asked to review existing educational/support materials e.g. leaflets and videos and provide feedback on, for example, language/phrasing, content, pitch and understanding. The research team will then facilitate cross-discussion between groups to develop the conclusions and a consensus.

In the final workshop draft intervention materials, developed from workshops 1 and 2, will be presented. For example, media channels that could be used to promote behaviour change such as testimonials, story-telling, and cooking demonstrations. The stakeholders will be divided into small groups to discuss and provide feedback on the acceptability of the components of the intervention and identify potential barriers to engagement. Following the small group discussions the researchers will facilitate feedback and encourage discussion as a whole to clarify/confirm the researcher’s interpretation. The intervention template may be further refined, and will be developed into the detailed programme.

For peer review only

For peer review only - http://bmjopen.bmj.com/site/about/guidelines.xhtml

Phase 2 – Evaluation of HEAL-D; a culturally-tailored T2D self-management programme for African and Caribbean communities

In phase 2 a feasibility study, with an embedded process evaluation, will be conducted to address the following objectives:

1. Evaluate the HEAL-D intervention, particularly its theoretical under-pinning, acceptability, fidelity, issues of implementation and sustainability.
2. Evaluate the feasibility of trial procedures, considering issues such as rates of recruitment, retention, completion and contamination.
3. Estimate the effect size of potential trial outcomes including HbA1c, weight, waist circumference, blood pressure, dietary intake, physical activity levels, diabetes knowledge, and quality of life, to inform an effectiveness trial.

Study Design

The feasibility study will use a randomised controlled design (RCT), with individual patients as the unit of randomisation, evaluating HEAL-D against usual care. In addition there will be a cohort of phase 1 co-design patients who will be allocated to the intervention arm (not randomised) because their involvement in the intervention design phase would contaminate the control arm. These patients will be included in the feasibility study to enable us to evaluate the impact of former involvement on intervention engagement, acceptability and ownership.

Participants

Participants will principally be recruited from General Practice in the London Boroughs of Lambeth and Southwark through screening of referrals for structured education and letters of invitation to patients with established T2D. In addition participants from the phase 1 co-design

For peer review only - http://bmjopen.bmj.com/site/about/guidelines.xhtml

study will be invited to participate, and self-referral methods will also be used, for example posters and advertisements in community locations.

Patients with diagnosed T2D who are of African or Caribbean ethnicity and with capacity to provide fully informed consent to participation in research will be eligible to participate in the trial. Ethnicity will be self-declared using the standard NHS ethnicity categorisation questionnaire. Patients who are unable to communicate in English and patients with complex therapeutic dietary needs may be ineligible to participate if their individual needs are deemed incompatible with the aims of the intervention. This is because the intervention will provide general diet and lifestyle advice for the self-management of T2D in a group setting; in cases of patients with certain comorbidities e.g. advanced renal disease, the intervention may be inappropriate for the individual, and the group nature of the intervention will prevent their individual needs from being addressed.

A pragmatic sample size of 120 patients is anticipated to be sufficient to evaluate the programme, allowing for 20% drop-out/non-completion; 80 patients will be randomised, 40 in each arm, and a further cohort of patients (n=40) from phase 1 will be allocated to the intervention arm without randomisation. As this is a feasibility trial it will not be powered to detect statistically significant intervention effects. A primary objective of the study is to provide estimates of key parameters such as potential effect sizes, recruitment and retention rates of the trial and participation rates of the programme, to enable the optimal design of a full-scale trial to be determined.

Intervention and control arms

Participants in the control arm will continue with usual care deemed appropriate and delivered by their primary care team, which may include referral to group structured education and/or one-to-one consultations with healthcare professionals.

For peer review only

Participants in the intervention arm will be offered the HEAL-D programme, which will deliver a curriculum of culturally-tailored, evidence-based diet and physical activity education and behavior change in a group setting. In line with clinical guidelines, the programme will be delivered by trained educators (external to the research team); favoured educators (e.g. lay educators *versus* healthcare professionals) will be identified in the co-design process. The details of each session, particularly the behavior change techniques and corresponding activities/materials will be identified through the co-design work.

The proposed curriculum will map to evidence-based guidelines, and will be as follows:

1. An introduction to T2D self-management principles.
2. Physical activity in T2D management.
3. Carbohydrates & portion sizes.
4. Weight management for T2D.
5. Managing cardiovascular health.

In line with clinical guidelines for diabetes structured education, the education sessions will be delivered through educator-led interactive discussion, however support materials will be provided to reinforce the learning, detailing evidence-based diet and physical activity guidance, which is culturally tailored for the African and Caribbean communities.

Data Collection

We will use a mixed methods approach, collecting a range of quantitative and qualitative data, to evaluate the intervention and the feasibility of trial procedures.

Estimating the effect of the intervention on potential trial outcomes

Participants will attend a baseline and post-intervention follow-up assessment visit, conducted by a research technician, at 26-32 weeks to collect the following potential trial outcomes and estimate effect sizes:

For peer review only - http://bmjopen.bmj.com/site/about/guidelines.xhtml

- HbA1c, total- HDL- & LDL-cholesterol, triglycerides: a 5ml venous blood sample will be taken for analysis of HbA1c & lipids.
- Body weight, height and body mass index (BMI): body weight will be measured using digital scales, with the patient wearing light clothing (without shoes), to the nearest 0.1 kg. Height will be measured, using a stadiometer, without shoes.
- Waist circumference: measured using a flexible tape, with the patient wearing only light clothing, at the mid-point between the lowest rib and the iliac crest.
- Systolic and diastolic blood pressure: the mean of three seated readings, taken using an automated sphygmomanometer.
- Diet & physical activity behaviours: dietary intake will be assessed through completion of a 24-hour diet recall, using the structured multiple pass interview method, and physical activity through 3-day Actiwatch accelerometer assessment and completion of the International Physical Activity Questionnaire (IPAQ).

The following validated self-complete questionnaires will be administered:

- Short Diabetes Knowledge Instrument (SDKI).
- Perceived Diabetes & Dietary Competence (PDDC).
- Diabetes Empowerment Scale- Short Form (DEC-SF).
- Multidimensional Scale of Perceived Social Support (PSS).
- Quality of life: EQ-5D-3L and PAID-5 questionnaires.

Evaluation of the HEAL-D intervention

Process evaluation is an essential part of testing complex interventions (39) and will be used in our feasibility trial to evaluate the HEAL-D intervention and the feasibility of trial procedures. Our process evaluation aims to achieve the following:

For peer review only

For peer review only - http://bmjopen.bmj.com/site/about/guidelines.xhtml

1. Test the intervention theory and whether the mechanisms of change operationalise as hypothesised.
2. Understand how the multiple components of the intervention interact.
3. Evaluate contextual factors that influence operationalisation of the intervention's theory/mechanisms of change, and any unintended effects of these factors.
4. Evaluate whether the intervention is differentiable from 'usual practice'.
5. Evaluate implementation of the intervention, particularly 'reach' (e.g. who receives the intervention), 'dose' and completion rates, and intervention fidelity (e.g. coverage of core materials and learning objectives during delivery, and the extent to which the programme is delivered in accordance with the delivery manual, what adaptations are undertaken and why).
6. Evaluate acceptability of the intervention to patients, healthcare professionals and commissioners.
7. Evaluate intervention embedding and sustainability e.g. what are the barriers and facilitators to the uptake of the intervention in current care pathways.

A range of quantitative and qualitative data will be collected, as detailed in Table 2. Attendance records, observation checklists, session/programme evaluation forms completed by patients and records of session activities completed by educators will provide quantitative data and will be used to evaluate a number of process domains, as indicated in Table 2. Our process evaluation will mainly focus on qualitative evaluations, with which we will use inductive reasoning to determine whether the intervention requires further development and adaptation. Patient interviews and focus groups, and interviews with educators, healthcare professionals and commissioners, and session observation notes will provide qualitative data for the evaluation of a number of process domains, as detailed in Table 2.

For peer review only

For peer review only - http://bmjopen.bmj.com/site/about/guidelines.xhtml

Evaluation of trial procedures

The feasibility of trial procedures will be evaluated, particularly rates and methods of recruitment, retention, completion, contamination between study arms and the proposed data collection methods:

Recruitment: a number of different pathways of recruitment will be implemented e.g. screening of primary care databases and letters of invitation, face-to-face referral during medical appointments, self-referral via posters, word-of-mouth referral. We will assess uptake rates from these different pathways to enable us to identify the most effectiveness methods and assess the feasibility of recruiting for a full-scale trial.

Retention & completion: we will assess the rate of retention both within the HEAL-D intervention (i.e. numbers completing each session and the full programme) and the feasibility trial (i.e. numbers completing baseline and endpoint assessment visits). We will evaluate the feasibility of randomising and retaining a control arm by assessing drop-out rates and comparing these between the study arms; we will also interview control arm patients to explore the acceptability of being assigned to the control arm.

Data collection methods: we will assess the frequency of missing data and any trends in which data is missing e.g. self-complete questionnaires, blood measures, to assess the feasibility of our data collection methods.

Contamination: we will interview patients from the control arm to explore issues of contamination e.g. did their participation in the trial promote change in self-management behaviours or motivate information-seeking behaviours, did they know anybody in the intervention arm or discuss the intervention with anybody.

For peer review only - http://bmjopen.bmj.com/site/about/guidelines.xhtml

PATIENT AND PUBLIC INVOLVEMENT

Service user involvement is intrinsic to this proposed research, which utilises participatory methods to engage patients and other stakeholders in the intervention design. The protocol provides extensive detail of how patients will be involved in the design, recruitment, conduct and dissemination of the research.

ETHICS & DISSEMINATION

The study protocol has been approved by the Fulham: London Research Ethics Committee (17-LO-1954); all participants will provide written consent prior to participation. All data will be anonymised and data protection protocols followed.

The study findings will be disseminated to the scientific community via conference presentations and peer-reviewed manuscripts, and to healthcare professionals via national and local clinical networks. The findings of the study will be communicated to our participants and local communities via the community networks and figureheads who we will engage in our participatory methods; we will give presentations at church events and publish a newsletter via our study website (www.heal-d.co.uk).

DISCUSSION

This paper presents the protocol for the design and feasibility testing of HEAL-D, a culturally-tailored T2D self-management programme for UK African and Caribbean communities. This study will employ rigorous complex intervention methodology to develop and evaluate the implementation of a culturally-tailored T2D self-management intervention. The intervention's curriculum will be based on existing evidence-based guidelines for diet and lifestyle management of T2D, participatory co-design methods will be employed to foster community engagement and partnership. We will use a 'bottom-up' approach to identify the cultural

For peer review only - http://bmjopen.bmj.com/site/about/guidelines.xhtml

adaptations of our intervention, and identify its theoretical basis through thematic analysis and the COM-B behavior change framework. The feasibility study will provide us with key information about the feasibility of running a full-scale trial of HEAL-D and process evaluation methods will enable us to understand how and why the intervention is effective or ineffective.

Culturally-tailored T2D education has been found to be more effective than standard education (40) but to date there have been no tailored education programmes for Black-British communities. A number of culturally-tailored diabetes education programmes have been developed for African-American communities; these have mainly used participatory methods to foster community engagement and have largely drawn on faith-based partnerships for their delivery (41, 42). It is not known to what extent these approaches and the content of these education programmes translates to the UK context, in which there are differences in both the healthcare systems and AfC culture to that of the USA. Indeed it is not known to what extent culturally-tailored care is needed for Black-British communities as little work has been undertaken with these communities. To date in the UK, culturally-tailored education programmes have been developed only for South Asian populations and other communities for whom English is not their first language, and 'tailoring' has focused on translating the delivery and resources into relevant languages. This type of adaptation would be considered a 'surface structure' in Resnicow's model of cultural tailoring (26). Our co-design work is intended to identify deeper levels of adaptation by exploring the socio-cultural barriers and facilitators to behaviour change and structuring HEAL-D accordingly. We acknowledge that we are likely to find huge diversity within our Black-British communities and *culture* will likely be only one of many important factors that affects their health behaviours. However, our co-design work will provide a more comprehensive theoretical under-pinning for the content of our programme than that which currently exists and will provide us with a framework upon which to evaluate

For peer review only - http://bmjopen.bmj.com/site/about/guidelines.xhtml

the effectiveness of our programme. This work will provide essential information and evaluation to inform the design of a future definitive trial.

For peer review only

For peer review only - http://bmjopen.bmj.com/site/about/guidelines.xhtml

ABBREVIATIONS

AfC	African-Caribbean
COM-B	Capability Opportunity Motivation Behaviour
HEAL-D	Healthy Eating & Active Lifestyles for Diabetes
MRC	Medical Research Council
NHS	National Health Service
T2D	Type 2 diabetes

DECLARATIONS

The study protocol has been approved by the Health Research Authority (London Fulham Research Ethics Committee; 17/LO/1954); all participants will provide written consent prior to participation.

CONSENT FOR PUBLICATION

Not applicable.

AVAILABILITY OF DATA AND MATERIALS

Not applicable.

COMPETING INTERESTS

The authors declare that they have no competing interests.

FUNDING

For peer review only - http://bmjopen.bmj.com/site/about/guidelines.xhtml

This report is independent research arising from a Career Development Fellowship (Louise M. Goff, CDF-2015-08-006) supported by the National Institute for Health Research. The views expressed in this publication are those of the author(s) and not necessarily those of the NHS, the National Institute for Health Research or the Department of Health.

AUTHOR CONTRIBUTIONS

All authors have made substantial contributions to this study. LMG, CR and SH were responsible for the conception and design of the study. LMG, CR, SH and AM developed the protocol and study approach. LMG drafted the manuscript. All authors read, revised and approved the final manuscript. LMG is guarantor.

ACKNOWLEDGEMENTS

Not applicable.

For peer review only

For peer review only - http://bmjopen.bmj.com/site/about/guidelines.xhtml

FIGURE LEGENDS

Figure 1. Medical Research Council's framework for the development and evaluation of complex interventions. Reproduced from Craig P. *et al.* British Medical Journal. 2008; 337:a1655.

Figure 2. Schematic diagram of Phase I: Development of HEAL-D using evidence synthesis and co-design methodology to design a culturally-tailored self-management programme for T2D in African and Caribbean communities

Figure 3. The Capability-Opportunity-Motivation (COM-B) Framework and Behaviour Change Wheel; a framework for developing behavioural interventions. Reproduced from Michie S., van Stralen M.M. and West R. Implementation Science. 2011; 6:42.

Figure 4. Applying the COM-B behaviour change framework to the development of the HEAL-D intervention; identifying theory of change.

For peer review only

For peer review only - http://bmjopen.bmj.com/site/about/guidelines.xhtml

Table 1. Topic guides for patient focus groups and stakeholder interviews

**Patient focus groups**	
	Knowledge and perceptions of diabetes, and diet and lifestyle advice for managing diabetes.
	Current practices relating to diabetes self-care, and diet and lifestyle.
	Health concerns/priorities in relation to diabetes.
	Motivations and barriers/difficulties relating to diabetes self-care, weight management and diet and lifestyle.
	Experiences and perceptions of diabetes care/education, and barriers to accessing care.
	Experiences of behaviour change in relation to diabetes, weight, diet and lifestyle – successes and failures.
	Role of family/friends/communities in influencing and shaping knowledge and behaviours in relation to diabetes, diet and lifestyle.
**Community leader interviews (including religious leaders)**	
	Health problems affecting the community and diabetes impact on health within this context.
	Attitude of the community towards health, medicines, doctors.
	Role of community leaders in promoting health and community activities.
	Diabetes health promotion activities within the community. What worked and what didn't.
	Barriers and facilitators to positive diabetes behaviours within the community.
	Advice about engaging the community: Who are the role models; What will engage and help people; How can healthcare & community work together.
**Healthcare professional interviews**	
	Experience of supporting African & Caribbean patients. What are the issues. How could things be improved. What factors make successful T2D management likely.
	Patient beliefs and motivations.
	Involvement in community activities and experience of working with community leaders and lay educators and suggestions to improve partnerships.
	Difficulties & challenges with offering a tailored lifestyle intervention.

For peer review only - http://bmjopen.bmj.com/site/about/guidelines.xhtml

Table 2. Mapping of the HEAL-D feasibility study research questions, process evaluation data sources and evaluation methods

PROCESS EVALUATION DOMAIN & RESEARCH QUESTIONS	DATA SOURCES: Patient questionnaires	Session observations	Session record of activities	Patient evaluation forms	Educator interviews	Patient interviews	Patient focus groups	Attendance records	HCP interviews	Commissioner interviews	EVALUATION METHOD
**TESTING INTERVENTION THEORY & MECHANISMS OF CHANGE**											
Are the intervention's mechanisms of change operationalised as hypothesised?	X	X	X	X	X	X	X				Qualitative data collected through interviews/focus groups with patients and educators, and session observation notes will be used to evaluate how the theory of the intervention operationalises and interacts with contextual factors.
How is the operationalisation of the mechanisms of change influenced by contextual factors?		X	X		X	X	X				
Does the interaction of the mechanisms of change with contextual factors give rise to unintended effects?		X	X		X	X	X				
**ASSESSING USUAL PRACTICE & CONTAMINATION**											
Is HEAL-D differentiable from 'usual practice'?						X					
Is there contamination in control patients?						X					Interviews will be conducted with patients from both arms. Experiences of the intervention and control will be explored. With control patients issues of contamination and perceptions of 'usual care' will be discussed.
**ASSESSING IMPLEMENTATION**											
What is the intervention reach and dose?	X							X			Questionnaire data will assess who receives the intervention and how representative they are e.g. age, gender, ethnicity, working status. Attendance records will be used to quantify the proportion of patients receiving the full *vs* part intervention.

For peer review only

Are the HEAL-D components/sessions delivered with fidelity and what is the nature of any adaptions?	X	X		X						To assess fidelity and compare intervention deliveries and contextual impacts educators will complete a record of activities & materials and list any resources/activities/discussions that were additional to the standardised schedule. These will be explored in depth in educator interviews which will be conducted at the end of the programme delivery. The research team will observe HEAL-D delivery to quantitatively assess coverage of curriculum, use of supporting materials and behaviour change techniques, quality of delivery, and participant engagement (binary score or a five-point Likert scale). Observers will qualitatively document course adaptations and general contextual observations.
Does the delivery of HEAL-D differ between sites, and what gives rise to differences?	X	X		X						
How well are the HEAL-D components/sessions delivered?	X	X								
**ASSESSING INTERVENTION ACCEPTABILITY**										
Is HEAL-D acceptable to patients, commissioners and healthcare professionals?	X		X		X	X		X	X	Acceptability will be evaluated through a range of qualitative and quantitative data. Quantitative data will be generated in patient evaluations, which will use 10-point scales to assess their views on the quality of the programme content, structure, format and delivery; the sessions/programme will be deemed ‘acceptable’ where they score ≥6 points. Interviews/focus groups with patients, educators, healthcare professionals and commissioners will explore acceptability through qualitative data e.g. reasons for attendance/non-attendance among patients, suggestions for amendments.
**ASSESSING INTERVENTION SUSTAINABILITY**										
How likely is the HEAL-D intervention to be sustainable and what factors might ensure sustainability?								X	X	Qualitative data collected through interviews with healthcare professionals and commissioners will be used to evaluate barriers & facilitators to implementation of HEAL-D into current care pathways, and its fit with organisational

For peer review only

For peer review only - http://bmjopen.bmj.com/site/about/guidelines.xhtml

		priorities, and the feasibility of sustained resource allocation to the HEAL-D intervention if found to be successful.

HCP, Healthcare professionals

For peer review only

For peer review only - http://bmjopen.bmj.com/site/about/guidelines.xhtml

REFERENCES

1. Estimating the current and future costs of Type 1 and Type 2 diabetes in the United Kingdom, including direct health costs and indirect societal and productivity costs [Internet]. York Health Economics Consortium Ltd. 2012.
2. Wilson C, Alam R, Latif S, Knighting K, Williamson S, Beaver K. Patient access to healthcare services and optimisation of self-management for ethnic minority populations living with diabetes: a systematic review. Health & social care in the community. 2012;20(1):1-19.
3. Becker E, Boreham R, Chaudhury M, Craig R, Deverill C, Doyle M, et al. Health Survey for England 2004. The health of minority ethnic groups. London: Joint Health Surveys Unit, National Centre for Social Research, Department of Epidemiology and Public Health at the Royal Free and University College Medical School; 2006.
4. Paul SK, Owusu Adjah ES, Samanta M, Patel K, Bellary S, Hanif W, et al. Comparison of body mass index at diagnosis of diabetes in a multi-ethnic population: A case-control study with matched non-diabetic controls. Diabetes, obesity & metabolism. 2017;19(7):1014-23.
5. Ng M, Fleming T, Robinson M, Thomson B, Graetz N, Margono C, et al. Global, regional, and national prevalence of overweight and obesity in children and adults during 1980-2013: a systematic analysis for the Global Burden of Disease Study 2013. Lancet. 2014;384(9945):766-81.
6. Lanting LC, Joung IM, Mackenbach JP, Lamberts SW, Bootsma AH. Ethnic differences in mortality, end-stage complications, and quality of care among diabetic patients: a review. Diabetes Care. 2005;28(9):2280-8.
7. James GD, Baker P, Badrick E, Mathur R, Hull S, Robson J. Type 2 diabetes: a cohort study of treatment, ethnic and social group influences on glycated haemoglobin. BMJ open. 2012;2(5).
8. Healthcare for London. Diabetes guide for London. 2009.
9. London Assembly. Review of diabetes care in London. 2013.
10. ONS. Census data 2011. 2011.
11. Leung G, Stanner S. Diets of minority ethnic groups in the UK: influence on chronic disease risk and implications for prevention. British Nutrition Bulletin. 2011;36:161-98.
12. Department of Health. Improving quality of life for people with long term conditions. London, UK: Department of Health; 2013.
13. Department of H. National service framework for diabetes standards2001.
14. National Institute for Health and Care Excellence. Type 2 diabetes in adults: management (NG28). 2015.
15. National Institute for Health and Care Excellence Quality and Outcomes Framework indicator. MN27: The percentage of patients newly diagnosed with diabetes, on the register, in the preceding 1 April to 31 March who have a record of being referred to a structured education programme within 9 months after entry on to the diabetes register. 2011.
16. Lakhani M. No patient left behind: how can we ensure world class primary care for black and minority ethnic people? London: Department of Health; 2008.
17. Healthcare Commission. Managing diabetes: improving services for people with diabetes. 2007.
18. Brown K, Avis M, Hubbard M. Health beliefs of African-Caribbean people with type 2 diabetes: a qualitative study. The British journal of general practice : the journal of the Royal College of General Practitioners. 2007;57(539):461-9.
19. Sarkar U, Piette JD, Gonzales R, Lessler D, Chew LD, Reilly B, et al. Preferences for self-management support: findings from a survey of diabetes patients in safety-net health systems. Patient education and counseling. 2008;70(1):102-10.
20. Stone MA. Making education in diabetes culturally appropriate for patients. Practice Nursing. 2006;17(12):5.
21. Cross T, Bazron B, Dennis K, Isaacs M. Towards a culturally competent system of care. Washington D.C.: Georgetown University Child Development Center, CASSP Technical Assistance Center; 1989.

For peer review only - http://bmjopen.bmj.com/site/about/guidelines.xhtml

22. Hawthorne K, Robles Y, Cannings-John R, Edwards AG. Culturally appropriate health education for type 2 diabetes mellitus in ethnic minority groups. The Cochrane database of systematic reviews. 2008(3):CD006424.
23. Liu JJ, Davidson E, Bhopal RS, White M, Johnson MRD, Netto G, et al. Adapting health promotion interventions to meet the needs of ethnic minority groups: mixed-methods evidence synthesis. Health Technol Assess. 2012;16(44).
24. Attridge M, Creamer J, Ramsden M, Cannings-John R, Hawthorne K. Culturally appropriate health education for people in ethnic minority groups with type 2 diabetes mellitus. Cochrane Database of Systematic Reviews. 2014(9):CD006424.
25. Matsumoto D. Culture and Psychology. Pacific Grove, CA.: Brooks/Cole.; 1996.
26. Resnicow K, Baranowski T, Ahluwalia JS, Braithwaite RL. Cultural sensitivity in public health: defined and demystified. Ethnicity & disease. 1999;9(1):10-21.
27. Dyson PA, Kelly T, Deakin T, Duncan A, Frost G, Harrison Z, et al. Diabetes UK evidence-based nutrition guidelines for the prevention and management of diabetes. Diabetic medicine : a journal of the British Diabetic Association. 2011;28(11):1282-8.
28. Medical Research Council. Developing and evaluating complex interventions: new guidance. 2006.
29. Di Noia J, Furst G, Park K, Byrd-Bredbenner C. Designing culturally sensitive dietary interventions for African Americans: review and recommendations. Nutrition reviews. 2013;71(4):224-38.
30. O'Mara-Eves A, Brunton G, McDaid D, Oliver S, Kavanagh J, Jamal F, et al. Community engagement to reduce inequalities in health: a systematic review, meta-analysis and economic analysis. Public Health Res. 2013;1(4).
31. Norris SL, Nichols PJ, Caspersen CJ, Glasgow RE, Engelgau MM, Jack L, et al. Increasing diabetes self-management education in community settings. A systematic review. American journal of preventive medicine. 2002;22(4 Suppl):39-66.
32. National Institute for Health and Care Excellence. Behaviour change: individual approaches. 2014. Report No.: Public health guidelines (PH49).
33. Wolfe WA. A review : maximizing social support-a neglected strategy for improving weight management with African-American women. Ethn Dis. 2004;14:212-8.
34. Kumanyika SK, Wadden TA, Shults J, et al. Trial of family and friend support for weight loss in african american adults. Archives of Internal Medicine. 2009;169(19):1795-804.
35. Sumlin LL, Garcia AA. Effects of food-related interventions for African American women with type 2 diabetes. The Diabetes educator. 2012;38(2):236-49.
36. Samuel-Hodge CD, DeVellis RF, Ammerman A, Keyserling TC, Elasy TA. Reliability and validity of a measure of perceived diabetes and dietary competence in African American women with type 2 diabetes. The Diabetes educator. 2002;28(6):979-88.
37. Abubakari AR, Jones MC, Lauder W, Kirk A, Anderson J, Devendra D, et al. Ethnic differences and socio-demographic predictors of illness perceptions, self-management, and metabolic control of type 2 diabetes. International journal of general medicine. 2013;6:617-28.
38. Michie S. Designing and implementing behaviour change interventions to improve population health. Journal of health services research & policy. 2008;13 Suppl 3:64-9.
39. Moore GF, Audrey S, Barker M, Bond L, Bonell C, Hardeman W, et al. Process evaluation of complex interventions: Medical Research Council guidance. BMJ. 2015;350:h1258.
40. Attridge M, Creamer J, Ramsden M, Cannings-John R, Hawthorne K. Culturally appropriate health education for people in ethnic minority groups with type 2 diabetes mellitus. The Cochrane database of systematic reviews. 2014(9):CD006424.
41. Two Feathers J, Kieffer EC, Palmisano G, Anderson M, Sinco B, Janz N, et al. Racial and Ethnic Approaches to Community Health (REACH) Detroit partnership: improving diabetes-related outcomes among African American and Latino adults. American journal of public health. 2005;95(9):1552-60.

For peer review only

42. Samuel-Hodge CD, Keyserling TC, Park S, Johnston LF, Gizlice Z, Bangdiwala SI. A Randomized Trial of a Church-Based Diabetes Self-management Program for African Americans With Type 2 Diabetes. Diabetes Educator. 2009;35(3):439-54.

For peer review only

For peer review only - http://bmjopen.bmj.com/site/about/guidelines.xhtml

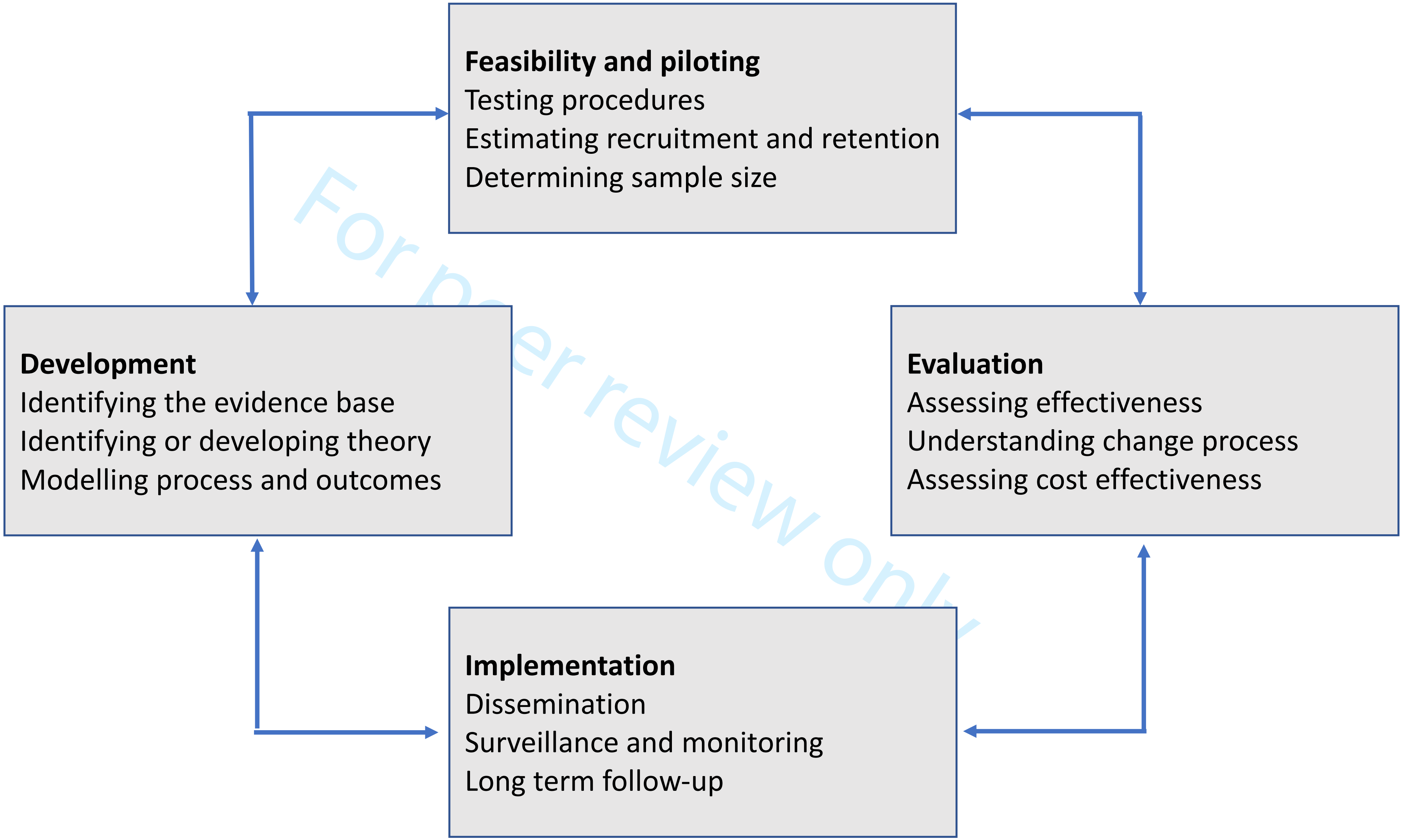

For peer review only - http://bmjopen.bmj.com/site/about/guidelines.xhtml

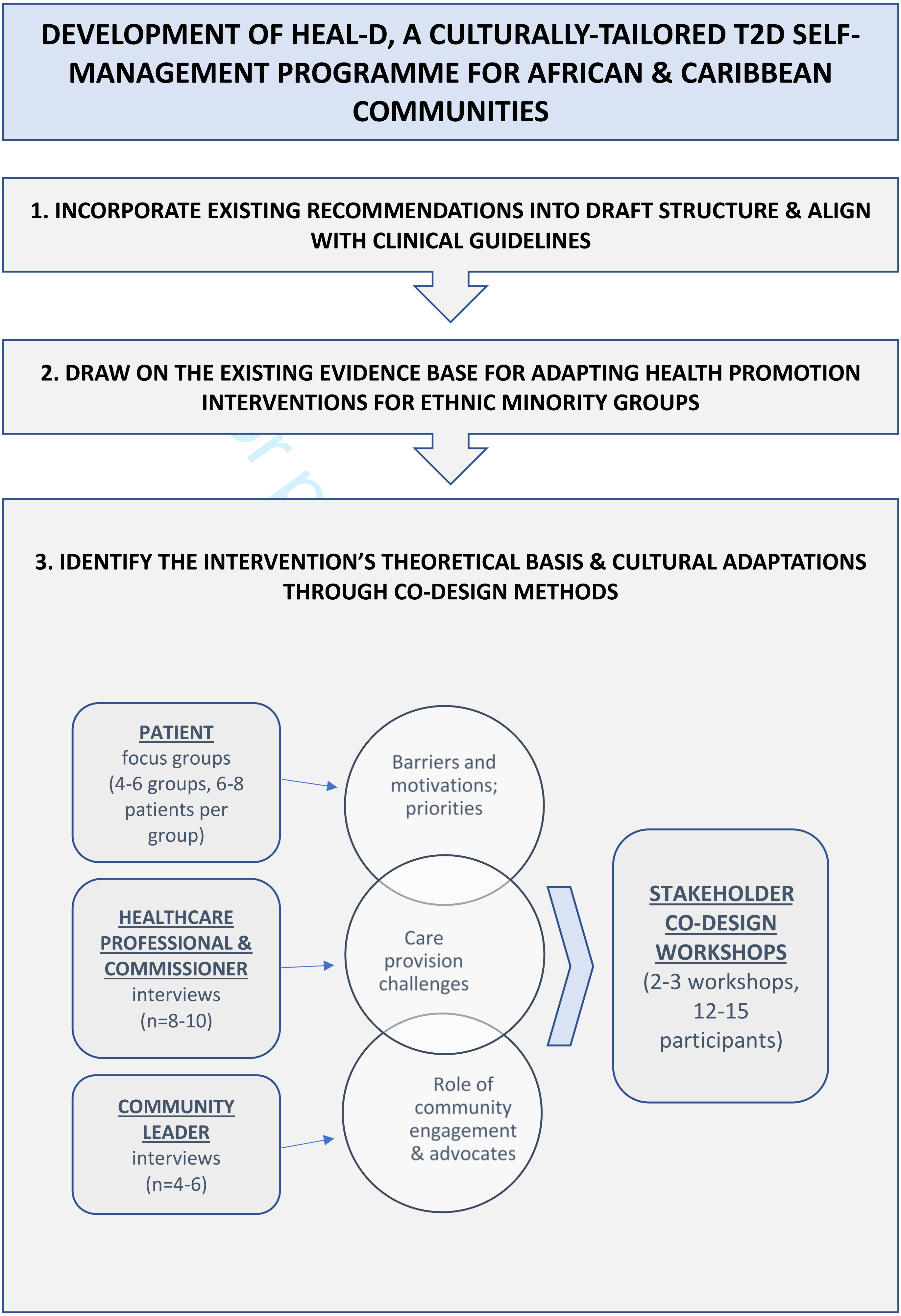
DEVELOPMENT OF HEAL-D, A CULTURALLY-TAILORED T2D SELF-MANAGEMENT PROGRAMME FOR AFRICAN & CARIBBEAN COMMUNITIES
1. INCORPORATE EXISTING RECOMMENDATIONS INTO DRAFT STRUCTURE & ALIGN WITH CLINICAL GUIDELINES
2. DRAW ON THE EXISTING EVIDENCE BASE FOR ADAPTING HEALTH PROMOTION INTERVENTIONS FOR ETHNIC MINORITY GROUPS
3. IDENTIFY THE INTERVENTION'S THEORETICAL BASIS & CULTURAL ADAPTATIONS THROUGH CO-DESIGN METHODS
PATIENT focus groups (4-6 groups, 6-8 patients per group)
HEALTHCARE PROFESSIONAL & COMMISSIONER interviews (n=8-10)
COMMUNITY LEADER interviews (n=4-6)
Barriers and motivations; priorities
Care provision challenges
Role of community engagement & advocates
STAKEHOLDER CO-DESIGN WORKSHOPS (2-3 workshops, 12-15 participants)

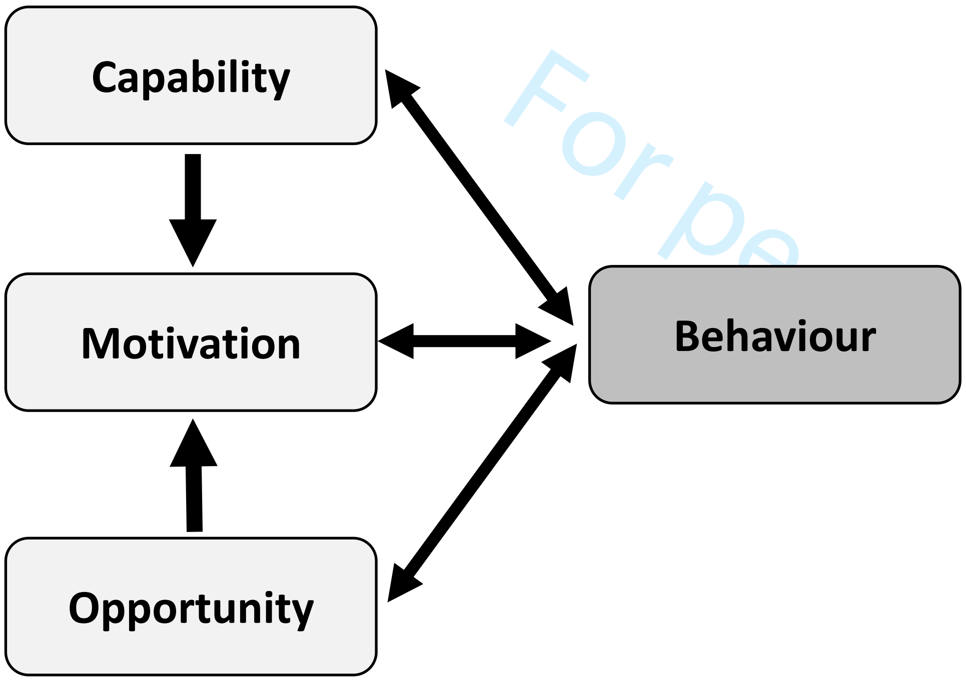
Capability
Motivation
Opportunity
Behaviour

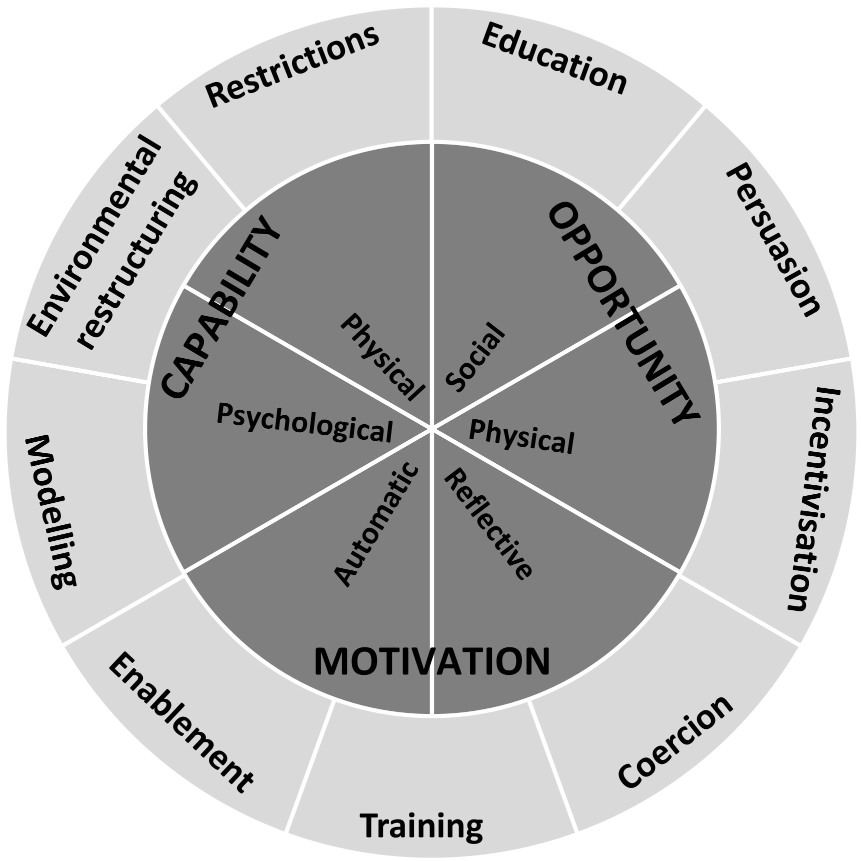
Restrictions
Education
Environmental restructuring
Persuasion
Modelling
Incentivisation
Enablement
Training
Coercion
CAPABILITY
OPPORTUNITY
MOTIVATION
Physical
Psychological
Social
Physical
Automatic
Reflective

COM-B analysis of the behaviour goal: performing 30 minutes moderate intensity physical activity per day

CAPABILITY

Knowledge: Does the target group know:

-Why moderate intensity physical activity would help diabetes management?

-What moderate intensity is and how to measure it?

Behavioural regulation: Does the target group know how to:

-Plan to fit the activity in to their daily life?

-Remember to do the activity?

-Prioritise this activity over others?

-Record & measure and self-monitor their activity?

Physical skills: Does the target group:

Have the physical stamina to be active at this intensity?

OPPORTUNITY

Environmental context & resources:

-Is it safe to exercise in the neighbourhood?

-Do patients have suitable footwear?

-Can they afford a pedometer or some means of measuring their activity?

Social influences (what interpersonal influences cause individuals to change their thoughts, feelings or behaviours?)

-It is culturally acceptable to exercise?

-What is the social norm among immediate friends and family?

-What positive or negative views do others have that may influence activity?

-Are there any positive role models?

-Are there competing demands e.g. pressure to spend leisure time with family or at church?

Would group support be motivating?

MOTIVATION

Reflective (conscious) motivation:

-How optimistic do the patients feel they can achieve the goal?

-Do they intend to do the behaviour (stages of change model)?

-What emotions may help or hinder? e.g. fear of injury.

-What other emotions may conflict? e.g tiredness, depression, stress.

Automatic (innate drivers):

-What are established habit patterns?

-What are routines/thought/behaviours set up by previous experience

Healthy Eating and Active Lifestyles for Diabetes (HEAL-D): study protocol for the design and feasibility trial, with process evaluation, of a culturally-tailored diabetes self-management programme for African-Caribbean communities

Journal:	*BMJ Open*
Manuscript ID	bmjopen-2018-023733.R2
Article Type:	Protocol
Date Submitted by the Author:	18-Dec-2018

Complete List of Authors:	Goff, Louise; Kings College Lonodn, Diabetes and Nutritional Sciences<br>Moore, Amanda; Kings College Lonodn, Diabetes and Nutritional Sciences<br>Rivas, Carol; University of Southamptom, Faculty of Health Sciences<br>Harding, Seeromanie; King\'s College London Division of Diabetes and Nutritional Sciences, Diabetes & Nutritional Sciences Division

**Primary Subject Heading**:	Diabetes and endocrinology
Secondary Subject Heading:	Nutrition and metabolism
Keywords:	type 2 diabetes, ethnicity, culture, diet, lifestyle, education

SCHOLARONE™
Manuscripts

Healthy Eating and Active Lifestyles for Diabetes (HEAL-D): study protocol for the design and feasibility trial, with process evaluation, of a culturally-tailored diabetes self-management programme for African-Caribbean communities

Louise M. Goff[1#], Amanda P. Moore[1], Carol Rivas[2], Seeromanie Harding[1]

[1]King's College London, Departments of Diabetes & Nutritional Sciences, School of Life Course Sciences, Faculty of Life Sciences & Medicine, [2]Institute of Education, University College London

[#]Corresponding author: Dr Louise M. Goff, Diabetes Research Group, King's College London, Franklin-Wilkins Building, Room 3.87, Waterloo Campus, King's College London, London SE1 9NH. T: 020 7848 4273; E: louise.goff@kcl.ac.uk

For peer review only - http://bmjopen.bmj.com/site/about/guidelines.xhtml

ABSTRACT

Introduction

Black-British communities are disproportionately burdened by type 2 diabetes (T2D) and its complications. Tackling these inequalities is a priority for healthcare providers and patients. Culturally-tailored diabetes education provides long-term benefits superior to standard care but to date such programmes have only been developed in the USA. The current programme of research aims to develop the Healthy Eating and Active Lifestyles for Diabetes (HEAL-D) culturally-tailored T2D self-management programme for Black-British communities; and to evaluate its delivery, acceptability and the feasibility of conducting a future effectiveness trial of HEAL-D.

Methods & analysis

Informed by Medical Research Council Complex Interventions guidance this research will rigorously develop and evaluate the implementation of the HEAL-D intervention to understand the feasibility of conducting a full-scale effectiveness trial. In Phase 1 the intervention will be developed. The intervention curriculum will be based on existing evidence-based T2D guidelines for diet and lifestyle management; co-design methods will be used to foster community engagement, identify the intervention's underpinning theory; identify the optimal structure, format and delivery methods, ascertain adaptations that are needed to ensure cultural sensitivity, and understand issues of implementation. In Phase 2 the intervention will be delivered and compared to usual care in a feasibility trial. Process evaluation methods will evaluate the delivery and acceptability of HEAL-D. The effect size of potential primary outcomes, such as HbA1c and body weight will be estimated. The feasibility of conducting a future effectiveness trial will also be evaluated, particularly feasibility of randomisation, recruitment, retention, and contamination.

Ethics & dissemination

For peer review only

This study is funded by a National Institute of Health Research Fellowship (CDF-2015-08-006), and approved by NHS Research Ethics Committee (17-LO-1954). Dissemination will be through national and international conferences, peer-reviewed publications and local and national clinical diabetes networks.

Trial Registration: this trial is registered with www.clinicaltrials.gov, identifier: NCT03531177.

STRENGTHS AND LIMITATIONS OF THIS STUDY

- This study employs rigorous complex intervention methodology to develop and evaluate a culturally-tailored diabetes self-management intervention.
- Participatory co-design methods are being used to foster stakeholder engagement in intervention development.
- The COM-B behaviour change framework is being used to identify appropriate intervention behaviour change techniques.
- Process evaluation measures are being collected to assess the feasibility of evaluating the intervention in a full-scale trial.
- The feasibility trial is designed to estimate the effect size of the intervention rather than efficacy, which will be the focus of a future definitive trial.

For peer review only

Keywords: African, Caribbean, ethnicity, type 2 diabetes, education, self-management, diet, lifestyle

For peer review only - http://bmjopen.bmj.com/site/about/guidelines.xhtml

INTRODUCTION

Type 2 diabetes (T2D) affects approximately 3 million people in England and consumes around 10% of the National Health Service (NHS) budget, estimated at almost £9 billion in 2011 and predicted to rise to 17% of the NHS budget by 2035 (1). Diabetes and its associated complications place an illness burden on patients and carers, which disproportionately affects those from ethnic minority backgrounds (2). The estimated prevalence of T2D is up to 3 times higher for Black-British communities compared to White Europeans (3). T2D occurs, on average, 10 years earlier in Black-British people, the mean age of diagnosis is 48 years and approximately 25% of patients are under the age of 40 years (4). Furthermore, glycaemic control is worse at the time of diagnosis, requires greater medical management, and poorer outcomes are evident (5-7). The reasons for these disparities are not fully understood; while biological factors are involved, it is understood that a range of behavioural, lifestyle and health system factors play a role. Tackling these inequalities is a healthcare priority (8, 9).

Individuals of Black-British ethnicity form the second largest ethnic minority population in the United Kingdom (UK); around 4% of the population self-identify from this ethnic background (10). Around half of individuals are of Black African ancestry and a third of Black Caribbean ancestry (10). Growth in the Black-British communities is relatively recent, beginning mainly in response post-second world war appeals to citizens of the Commonwealth regions to assist with gaps in its labour market. This prompted a large influx of migrants in the 1950s from the Caribbean islands, particularly Jamaica. Migration from the African continent has been more recent, peaking around the 1980s; migrants from African nations currently form the largest growing ethnic minority group in the UK population (11). In some regions, such as London, Black-British communities may represent 30-40% of the local population and are therefore a 'majority-minority' community. Other demographic patterns are recognised; the age distribution of the Black African and Black Caribbean communities differs, with a larger

For peer review only - http://bmjopen.bmj.com/site/about/guidelines.xhtml

proportion of Black Caribbeans being aged 65 years and over, while in the Black African population a greater proportion are children and young adults. High rates of unemployment are evident, averaging around 12% compared to 4% in the White British population (11).

Poor access to diabetes healthcare is a significant issue for minority ethnic groups (2). In the UK the NHS provides care to all UK residents that is free at the point of delivery. First-line diabetes management is situated in primary care and aims to promote patient involvement and self-management (12), enabling patients to adopt a healthy lifestyle and to manage their diabetes through support and education (13). To achieve this, UK T2D management guidelines recommend that all patients attend a structured education course to teach them the principals of T2D self-management and that this be offered annually from the time of diagnosis (14). Courses are recommended to use a group structure; typically they use face-to-face delivery by a diabetes specialist nurse or dietitian, with lay educator co-delivery in some cases (14). Referral to such courses is audited and incentivised through the Quality Outcomes Framework (15). Ethnic minority groups report finding it more difficult to access primary care services (16) and are more likely to report that they have not had the opportunity to attend a diabetes education course than White populations (17). Specifically, African-Caribbean (AfC) communities often report a distrust of medical advice and a desire for natural, non-pharmacological therapies (18). Furthermore, healthcare professionals are perceived as lacking cultural understanding (19) and their advice as lacking cultural relevance (20) or being poorly adapted to culture and needs (18), despite their intentions; these issues may contribute to the poorer diabetes outcomes and increased morbidity experienced by AfC patients.

Culturally tailored healthcare is proposed to be one of the main ways in which healthcare disparities can be addressed (21-23) and is identified as a priority by patients (8). Culturally-tailored diabetes education has demonstrated greater improvements in diabetes control and knowledge than usual care, and the benefits are maintained long-term (22, 24). Culture is a

For peer review only - http://bmjopen.bmj.com/site/about/guidelines.xhtml

concept that is notoriously difficult to define but generally within healthcare it is thought of as ‘a set of attitudes, values, beliefs and behaviours shared by a group of people, communicated from one generation to the next’ (25). In their model for understanding cultural sensitivity in healthcare, Resnicow *et al.* (1999) described two dimensions in culture: surface and deep structures. Tailoring interventions to surface structures involves matching materials and messages to observable, "superficial" characteristics of a target population e.g. language and food, familiar to, and preferred by, the target audience. Deep structure involves incorporating the cultural, social, historical, environmental and psychological forces that influence the target health behaviours in the proposed target population. Whereas surface structure generally increases the "receptivity" or "acceptance" of messages, deep structure conveys salience (26). Culture is ever evolving for any group and it is important to recognise the diversity that exists within any one ‘cultural group’, which is particularly evident in migrant populations where second/third generations may have undergone significant acculturation. To date, culturally tailored interventions for the African diaspora have largely been based in the USA, and may not translate to UK healthcare structures or UK AfC communities whose cultural needs may be different (23).

A two-phase programme of research is proposed in which a culturally-tailored, evidence-based self-management programme for T2D in African and Caribbean communities, called Healthy Eating & Active Lifestyles for Diabetes (HEAL-D), is developed, followed by a feasibility trial. The intervention curriculum will be based on existing evidence-based guidelines for T2D (14, 27) to enable it to have potential to be embedded into clinical practice; co-design methods will be used to identify the optimal structure, format and methods of delivery and to ascertain appropriate adaptations that are needed to ensure cultural sensitivity of the content. The purpose of this article is to present the protocol for the development and feasibility trial of HEAL-D.

For peer review only

For peer review only - http://bmjopen.bmj.com/site/about/guidelines.xhtml

PURPOSE & AIMS

The overall aims of this research are to develop a culturally-tailored, evidence-based self-management programme for managing T2D among AfC communities in primary care, called HEAL-D, and to determine the feasibility of evaluating HEAL-D through a future effectiveness trial.

The objectives are to:

1. Develop a self-management programme, based on existing evidence-based diet and lifestyle guidelines, appropriately tailored for AfC patients through co-design methods.
2. Establish the feasibility of conducting an effectiveness trial of HEAL-D, considering issues such as participation rates and potential effect sizes.

For peer review only

For peer review only - http://bmjopen.bmj.com/site/about/guidelines.xhtml

METHODS AND ANALYSIS

Guided by the Medical Research Council's Complex Interventions framework (28) (Figure 1), two distinct phases of research are proposed: phase 1 is a formative phase in which the HEAL-D intervention will be developed; and phase 2 will evaluate the HEAL-D intervention in a feasibility trial. Study recruitment began in April 2017; the study duration will be 36 months.

Phase 1 – Development of a culturally-tailored T2D self-management programme

The process for the development of HEAL-D is outlined in Figure 2. Firstly, to ensure its potential to be embedded into future clinical practice, the HEAL-D curriculum will align with existing UK management recommendations and guidelines published by the National Institute of Clinical Excellence and Diabetes UK (14, 27):

Guidelines for diet and lifestyle management of T2D (27):

1. Achieve 5-10% weight loss or weight maintenance in those of healthy weight.
2. Undertake 150 minutes/week of moderate-to-vigorous intensity aerobic physical activity plus 2 sessions/week of strength training.
3. Balance carbohydrate intakes through portion control and promotion of low glycaemic index and wholegrain sources.
4. Limit saturated fat intake (<10% of energy intake), replace with mono-unsaturated fats.
5. Limit salt intake (<6g per day).
6. Consume oily fish at least twice per week.

Guidelines for T2D recommend that self-management structured education is offered to adults with T2D and/or their family members or carers, with group education as the preferred option, and that the education programmes are theory-driven, evidence-based and meet the cultural, linguistic, cognitive and literacy needs of the population (14).

For peer review only - http://bmjopen.bmj.com/site/about/guidelines.xhtml

Drawing on the existing evidence base

Secondly it will draw on key themes reported in published literature relating to methodologies for adapting health promotion interventions for ethnic minority groups. These have been evaluated in a number of recent systematic reviews; aside from acknowledging the lack of UK-based studies, these reviews make several recommendations. The powerful influence of social networks on health beliefs and behaviours should be acknowledged (29), and a focus on community-level interventions should be taken; delivering care in a social context promotes engagement and has been shown to be more effective than traditional individual-centred behavioural approaches (23). Community engagement should be promoted to overcome issues of deep-rooted, historical distrust of medical advice and settings, to develop and nurture trust between the researchers and community, and to nurture the strong sense of collectivism and kinship networks that are evident amongst AfC communities. Participatory methods (e.g. patient involvement in intervention design, lay-led delivery of interventions) should be employed as they are highly effective at improving health behaviours and self-efficacy across a number of conditions (30). Using community gathering places (e.g. faith institutions) as intervention settings offers the benefit of cultural relevancy and may reach populations who would not normally access self-management education (31).

Identifying the intervention's theoretical basis

Behavioural interventions should have a theoretical under-pinning (28, 32) so that the changes that are expected, and how these will be achieved, can be predicted from consideration of known behaviour change techniques. While there have been a number of interventions tailored to support diet and lifestyle behaviour change in AfC communities (33), their theoretical underpinning has rarely been drawn out or clearly presented. The theoretical underpinning of

For peer review only - http://bmjopen.bmj.com/site/about/guidelines.xhtml

HEAL-D will be developed through a combination of key themes from the published literature and new primary research.

In the literature collectivism and the importance of social interaction for people of AfC ancestry is well reported (29), and the provision of a social support group, or inclusion of a family member, has been shown to be particularly effective in lifestyle interventions in African-American communities (34, 35). These findings suggest social learning theory, which focuses on promoting behaviour change through social interaction, role modelling and social comparison, may be a relevant behaviour change theory for our intervention. Notably, much of literature that identifies the drivers of health behaviours in AfC communities and may, therefore, inform the theoretical basis of an intervention, comes from the USA and it is not known to what extent these findings apply to AfC in other regions. One of the reasons we will use co-design methods will be to understand the relevance of these existing themes to the UK context and enable us to identify themes that are important to Black-British communities.

Co-designing the intervention through participatory methods

HEAL-D will use participatory co-design methods to engage patients, healthcare providers and community leaders (e.g. church leaders, community group leads) in focus groups, interviews and workshops to achieve the following:

1. Foster community engagement.
2. Identify the theoretical under-pinning of HEAL-D and its mechanisms of action.
3. Identify appropriate cultural adaptations for the intervention.
4. Understand issues of intervention implementation.

Focus groups and interviews

Focus groups, 8-10 groups of 6-8 participants, will be conducted with patients with T2D of AfC ethnicity, recruited through local churches, mosques and community groups, as well as

For peer review only - http://bmjopen.bmj.com/site/about/guidelines.xhtml

through GP practices in London. The focus groups will be conducted in local accessible community venues e.g. church hall, library, community centre. Patients will be purposively sampled to get a spread of socio-economic position, generational status and ancestral origins, as principal factors impacting on health status, healthcare access and cultural behaviours in these groups (36-38). Separate focus groups will be conducted with men and women, and patients of direct African *versus* Caribbean ancestry, as they report different cultural barriers/facilitators to lifestyle change (36, 37). A topic guide (Table 1) based on themes identified in the literature, will be used to steer discussions and ensure coverage of key themes whilst encouraging free discussion of opinion/perspective. Focus groups have been selected to enable us to understand normative needs, as suited to the development of a community intervention.

Semi-structured interviews will be conducted with 8-10 healthcare providers, including general practitioners, practice nurses, diabetes specialist nurses, diabetes specialist dietitians and commissioners. The interviews will cover issues relating to healthcare needs and engagement of AfC patients, experiences of delivering healthcare to AfC patients, and barriers and facilitators to working in partnership with community groups to deliver care for AfC communities (Table 1). Interviews have been selected for this part of the study to enable us to gather a full range of experiences and therefore optimise implementation.

Community leaders representing faith and non-faith institutions (n=4-6) will be invited to participate in semi-structured interviews. Leaders will be identified initially through existing networks e.g. Diabetes UK Community Champions initiative. Word-of-mouth and ‘snow-balling’ techniques that are highly effective within these communities, will be used to recruit a wider network. The interviews will cover issues relating to the role of community networks in promoting health of AfC communities, sustaining health amongst community members, and opportunities for greater impact (Table 1).

For peer review only - http://bmjopen.bmj.com/site/about/guidelines.xhtml

Analysis

The focus groups and interviews will be digitally recorded and transcribed verbatim. The data will be analysed using the framework approach in NVivo (QSR International), theoretically driven by socio-ecological theory to identify themes relating to issues at the individual, family, community and healthcare delivery levels and how these influence self-efficacy and behaviour change. Our analysis will identify priority behaviours of focus for the intervention, key barriers and facilitators to behaviour change and healthcare engagement, favoured settings, and a rudimentary draft of the cultural adaptations. Deviant case analysis, that is consideration of cases that do not fit the general picture, will be undertaken, though our primary interest is in the commonalities as this is a community level intervention. Primary coding and development of a coding scheme will be carried out by a single researcher; a second researcher will independently use this coding scheme to code 20% of the data for cross-comparison, to improve dependability. This will provide methodological rigour required for confidence in the analysis of the qualitative data. The themes will be fed-back and discussed with a Service User Group (SUG), which will consist of representatives of patients, healthcare providers, and community leaders. The SUG will be set up to inform and guide each stage of the research plan and will be a forum through which the research team can seek the opinion of key stakeholders, in this case particularly relating to interpretation of the qualitative data and to ensure trustworthiness of conceptualisations. The SUG will also review research documents, such as patient information sheets and questionnaires, and provide feedback on their content and suitability for the communities of focus.

We will divide our data into behavioural ‘barriers’ and ‘facilitators’ where possible. To ascertain appropriate behaviour change techniques for our intervention (32) we will map our analysis onto the Capability-Opportunity-Motivation-Behaviour (COM-B) framework from the Behaviour Change Wheel (39) (Figure 3), and thence in each case consider the outcome

For peer review only - http://bmjopen.bmj.com/site/about/guidelines.xhtml

behaviours that our intervention will aim to achieve, a worked example is shown in Figure 4. We will use the COM-B framework to identify appropriate *functions* of our intervention to optimise facilitators and overcome barriers to achievement of planned outcomes, e.g. 'education' for capability barriers, 'modelling' for opportunity and motivation barriers. Finally, we will select specific behaviour change techniques e.g. education, goal setting, that focus on the specific functions we have identified. We will also look to identify other themes that arise from the data, which might not map clearly onto the COM-B framework (e.g. contextual themes relating to the health system) but which may inform our intervention theory as well as help us to understand issues of implementation (e.g. favoured settings and timings). Through this analysis we will identify our intervention theory that we will draw on for the next stage of the study, as documented through a logic diagram.

Stakeholder co-design workshops

Following evaluation of the focus groups and interviews our stakeholders, 12-15 patients, healthcare providers, commissioners and community leaders, will be invited to participate in a series of 2-3 half-day workshops, held in community locations. The workshops will seek to gain stakeholder involvement in developing the details of the interventions. This will include determining the setting, the media channels, structure and delivery, as well as steering the research team to understand and respond to literacy and numeracy needs. The workshops will endeavour to reach a consensus opinion from attendees but where stakeholders have different needs and a consensus cannot be reached the research team will consult with the SUG to make decisions on the way forward and consider where there is scope for the intervention to be structured to meet these different needs e.g. delivery in a range of settings. In the first workshop the research team will feed back the findings of the focus groups and interviews; anonymised interview extracts will be presented to illustrate the key themes and issues that were identified. The stakeholders will be asked to discuss the themes and behavioural targets in small groups,

For peer review only - http://bmjopen.bmj.com/site/about/guidelines.xhtml

using directed tasks/questions to facilitate the discussions. Following the small group discussions the researchers will facilitate discussion as a whole to clarify/confirm interpretation; open discussion/debate will be encouraged to examine the themes in depth and for all stakeholders to agree a mutual understanding.

In the second workshop elements of the proposed intervention will be presented for comment, refining and development. Using scenarios, the stakeholders will be asked to brainstorm, in small groups, key issues relating to the scenarios. For example, the moderator will present scenarios relating to the intervention setting and the attendees will be asked to discuss and identify the pros and cons of each, and then feedback their discussions to the other attendees. The attendees will be asked to review existing educational/support materials e.g. leaflets and videos and provide feedback on, for example, language/phrasing, content, pitch and understanding. The research team will then facilitate cross-discussion between groups to develop the conclusions and a consensus.

In the final workshop draft intervention materials, developed from workshops 1 and 2, will be presented. For example, media channels that could be used to promote behaviour change such as testimonials, story-telling, and cooking demonstrations. The stakeholders will be divided into small groups to discuss and provide feedback on the acceptability of the components of the intervention and identify potential barriers to engagement. Following the small group discussions the researchers will facilitate feedback and encourage discussion as a whole to clarify/confirm the researcher's interpretation. The intervention template may be further refined, and will be developed into the detailed programme.

Phase 2 – Evaluation of HEAL-D; a culturally-tailored T2D self-management programme for African and Caribbean communities

For peer review only

In phase 2 a feasibility study, with an embedded process evaluation, will be conducted to address the following objectives:

1. Evaluate the HEAL-D intervention, particularly its theoretical under-pinning, acceptability, fidelity, issues of implementation and sustainability.
2. Evaluate the feasibility of trial procedures, considering issues such as rates of recruitment, retention, completion and contamination.
3. Estimate the effect size of potential trial outcomes including HbA1c, weight, waist circumference, blood pressure, dietary intake, physical activity levels, diabetes knowledge, and quality of life, to inform an effectiveness trial.

Study Design

The feasibility study will use a randomised controlled design (RCT), with individual patients as the unit of randomisation, evaluating HEAL-D against usual care. In addition, there will be a cohort of phase 1 co-design patients who will be allocated to the intervention arm (not randomised) because their involvement in the intervention design phase would contaminate the control arm. These patients will be included in the feasibility study to enable us to evaluate the impact of former involvement on intervention engagement, acceptability and ownership.

Participants

Participants will principally be recruited from General Practice in the London Boroughs of Lambeth and Southwark through screening of referrals for structured education and letters of invitation to patients with established T2D. In addition, participants from the phase 1 co-design study will be invited to participate, and self-referral methods will also be used, for example posters and advertisements in community locations.

Patients with diagnosed T2D who are of African or Caribbean ethnicity and with capacity to provide fully informed consent to participation in research will be eligible to participate in the

For peer review only - http://bmjopen.bmj.com/site/about/guidelines.xhtml

trial. Ethnicity will be self-declared using the standard NHS ethnicity categorisation questionnaire. Patients who are unable to communicate in English and patients with complex therapeutic dietary needs may be ineligible to participate if their individual needs are deemed incompatible with the aims of the intervention. This is because the intervention will provide general diet and lifestyle advice for the self-management of T2D in a group setting; in cases of patients with certain comorbidities e.g. advanced renal disease, the intervention may be inappropriate for the individual, and the group nature of the intervention will prevent their individual needs from being addressed.

A pragmatic sample size of 120 patients is anticipated to be sufficient to evaluate the programme, allowing for 20% drop-out/non-completion; 80 patients will be randomised, 40 in each arm, and a further cohort of patients (n=40) from phase 1 will be allocated to the intervention arm without randomisation. As this is a feasibility trial it will not be powered to detect statistically significant intervention effects. A primary objective of the study is to provide estimates of key parameters such as potential effect sizes, recruitment and retention rates of the trial and participation rates of the programme, to enable the optimal design of a full-scale trial to be determined.

Intervention and control arms

Participants in the control arm will continue with usual care deemed appropriate and delivered by their primary care team, which may include referral to group structured education and/or one-to-one consultations with healthcare professionals.

Participants in the intervention arm will be offered the HEAL-D programme, which will deliver a curriculum of culturally-tailored, evidence-based diet and physical activity education and behavior change in a group setting. In line with clinical guidelines, the programme will be delivered by trained educators (external to the research team); favoured educators (e.g. lay educators *versus* healthcare professionals) will be identified in the co-design process. The

For peer review only - http://bmjopen.bmj.com/site/about/guidelines.xhtml

details of each session, particularly the behavior change techniques and corresponding activities/materials will be identified through the co-design work.

The proposed curriculum will map to evidence-based guidelines, and will be as follows:

1. An introduction to T2D self-management principles.
2. Physical activity in T2D management.
3. Carbohydrates & portion sizes.
4. Weight management for T2D.
5. Managing cardiovascular health.

In line with clinical guidelines for diabetes structured education, the education sessions will be delivered through educator-led interactive discussion, however support materials will be provided to reinforce the learning, detailing evidence-based diet and physical activity guidance, which is culturally tailored for the African and Caribbean communities.

Data Collection

We will use a mixed methods approach, collecting a range of quantitative and qualitative data, to evaluate the intervention and the feasibility of trial procedures.

Estimating the effect of the intervention on potential trial outcomes

Participants will attend a baseline and post-intervention follow-up assessment visit, conducted by a research technician, at 26-32 weeks to collect the following potential trial outcomes and estimate effect sizes:

Biomedical outcomes: a 5ml venous blood sample will be taken for analysis of HbA1c and total- HDL- & LDL-cholesterol, triglycerides. Systolic and diastolic blood pressure will be measured using an automated sphygmomanometer.

Anthropometric outcomes: body weight will be measured using digital scales, with the patient wearing light clothing (without shoes); height will be measured, using a stadiometer, without

For peer review only - http://bmjopen.bmj.com/site/about/guidelines.xhtml

shoes; body mass index (BMI) will be calculated as [weight kg/height m^2]. Waist circumference will be measured with the patient wearing only light clothing, at the mid-point between the lowest rib and the iliac crest.

Diet & physical activity behaviour outcomes: dietary intake will be assessed through completion of a 24-hour diet recall, using the structured multiple pass interview method, and physical activity through 3-day Actiwatch accelerometer assessment and completion of the International Physical Activity Questionnaire (IPAQ).

The following validated self-complete questionnaires will be administered to assess: diabetes knowledge (Short Diabetes Knowledge Instrument (40)); diabetes and diet knowledge and competence (Perceived Diabetes & Dietary Competence (37)); empowerment (Diabetes Empowerment Scale- Short Form (41)); social support (Multidimensional Scale of Perceived Social Support (42)); diabetes distress (PAID-5 (43)) and quality of life (EQ-5D-3L (44)).

Statistical analysis: Given that this is a feasibility study with a small sample size, descriptive statistics will be used (Chi-Square test, Fisher's exact test). Differences between the groups in all outcomes will be estimated with 95% confidence intervals. The descriptive data will provide stable estimates of the variability of continuous outcomes by group, and provide estimates of differences between the groups in means and proportions for the key outcomes. The standard deviations of the mean change in HbA1c will be estimated by arms and used to derive the sample size calculation for a subsequent trial.

Evaluation of the HEAL-D intervention

Process evaluation is an essential part of testing complex interventions (45) and will be used in our feasibility trial to evaluate the HEAL-D intervention and the feasibility of trial procedures. Our process evaluation aims to achieve the following:

For peer review only - http://bmjopen.bmj.com/site/about/guidelines.xhtml

1. Test the intervention theory and whether the mechanisms of change operationalise as hypothesised.
2. Understand how the multiple components of the intervention interact.
3. Evaluate contextual factors that influence operationalisation of the intervention's theory/mechanisms of change, and any unintended effects of these factors.
4. Evaluate whether the intervention is differentiable from 'usual practice'.
5. Evaluate implementation of the intervention, particularly 'reach' (e.g. who receives the intervention), 'dose' and completion rates, and intervention fidelity (e.g. coverage of core materials and learning objectives during delivery, and the extent to which the programme is delivered in accordance with the delivery manual, what adaptations are undertaken and why).
6. Evaluate acceptability of the intervention to patients, healthcare professionals and commissioners.
7. Evaluate intervention embedding and sustainability e.g. what are the barriers and facilitators to the uptake of the intervention in current care pathways.

A range of quantitative and qualitative data will be collected, as detailed in Table 2. Attendance records, observation checklists, session/programme evaluation forms completed by patients and records of session activities completed by educators will provide quantitative data and will be used to evaluate a number of process domains, as indicated in Table 2. Our process evaluation will mainly focus on qualitative evaluations, with which we will use inductive reasoning to determine whether the intervention requires further development and adaptation. Patient interviews and focus groups, and interviews with educators, healthcare professionals and commissioners, and session observation notes will provide qualitative data for the evaluation of a number of process domains, as detailed in Table 2.

For peer review only

For peer review only - http://bmjopen.bmj.com/site/about/guidelines.xhtml

Evaluation of trial procedures

The feasibility of trial procedures will be evaluated, particularly rates and methods of recruitment, retention, completion, contamination between study arms and the proposed data collection methods:

Recruitment: several different pathways of recruitment will be implemented e.g. screening of primary care databases and letters of invitation, face-to-face referral during medical appointments, self-referral via posters, word-of-mouth referral. We will assess uptake rates from these different pathways to enable us to identify the most effective methods and assess the feasibility of recruiting for a full-scale trial.

Retention & completion: we will assess the rate of retention both within the HEAL-D intervention (i.e. numbers completing each session and the full programme) and the feasibility trial (i.e. numbers completing baseline and endpoint assessment visits). We will evaluate the feasibility of randomising and retaining a control arm by assessing drop-out rates and comparing these between the study arms; we will also interview control arm patients to explore the acceptability of being assigned to the control arm.

Data collection methods: we will assess the frequency of missing data and any trends in which data is missing e.g. self-complete questionnaires, blood measures, to assess the feasibility of our data collection methods.

Contamination: we will interview patients from the control arm to explore issues of contamination e.g. did their participation in the trial promote change in self-management behaviours or motivate information-seeking behaviours, did they know anybody in the intervention arm or discuss the intervention with anybody.

For peer review only - http://bmjopen.bmj.com/site/about/guidelines.xhtml

PATIENT AND PUBLIC INVOLVEMENT

Service user involvement is intrinsic to this proposed research, which utilises participatory methods to engage patients and other stakeholders in the intervention design. The protocol provides extensive detail of how patients will be involved in the design, recruitment, conduct and dissemination of the research.

ETHICS & DISSEMINATION

The study protocol has been approved by the Fulham: London Research Ethics Committee (17-LO-1954); all participants will provide written consent prior to participation. All data will be anonymised and data protection protocols followed.

The study findings will be disseminated to the scientific community via conference presentations and peer-reviewed manuscripts, and to healthcare professionals via national and local clinical networks. The findings of the study will be communicated to our participants and local communities via the community networks and figureheads who we will engage in our participatory methods; we will give presentations at church events and publish a newsletter via our study website (www.heal-d.co.uk).

DISCUSSION

This paper presents the protocol for the design and feasibility testing of HEAL-D, a culturally-tailored T2D self-management programme for UK African and Caribbean communities. This study will employ rigorous complex intervention methodology to develop and evaluate the implementation of a culturally-tailored T2D self-management intervention. The intervention's curriculum will be based on existing evidence-based guidelines for diet and lifestyle management of T2D, participatory co-design methods will be employed to foster community engagement and partnership. We will use a 'bottom-up' approach to identify the cultural

For peer review only - http://bmjopen.bmj.com/site/about/guidelines.xhtml

adaptations of our intervention, and identify its theoretical basis through thematic analysis and the COM-B behavior change framework. The feasibility study will provide us with key information about the feasibility of running a full-scale trial of HEAL-D and process evaluation methods will enable us to understand how and why the intervention is effective or ineffective.

To date there have been no tailored education programmes for Black-British communities. Indeed it is not known to what extent culturally-tailored care is needed for Black-British communities as little work has been undertaken with these communities. Our co-design work is intended to explore the socio-cultural barriers and facilitators to behaviour change and structure HEAL-D accordingly. We acknowledge that we are likely to find huge diversity within our Black-British communities and *culture* will likely be only one of many important factors that affects their health behaviours. However, our co-design work will provide a more comprehensive theoretical under-pinning for the content of our programme than that which currently exists and will provide us with a framework upon which to evaluate the effectiveness of our programme. This work will provide essential information and evaluation to inform the design of a future definitive trial.

For peer review only

For peer review only - http://bmjopen.bmj.com/site/about/guidelines.xhtml

ABBREVIATIONS

AfC	African-Caribbean
COM-B	Capability Opportunity Motivation Behaviour
HEAL-D	Healthy Eating & Active Lifestyles for Diabetes
MRC	Medical Research Council
NHS	National Health Service
T2D	Type 2 diabetes

DECLARATIONS

The study protocol has been approved by the Health Research Authority (London Fulham Research Ethics Committee; 17/LO/1954); all participants will provide written consent prior to participation.

CONSENT FOR PUBLICATION

Not applicable.

AVAILABILITY OF DATA AND MATERIALS

Not applicable.

COMPETING INTERESTS

The authors declare that they have no competing interests.

FUNDING

For peer review only

For peer review only - http://bmjopen.bmj.com/site/about/guidelines.xhtml

This report is independent research arising from a Career Development Fellowship (Louise M. Goff, CDF-2015-08-006) supported by the National Institute for Health Research. The views expressed in this publication are those of the author(s) and not necessarily those of the NHS, the National Institute for Health Research or the Department of Health.

AUTHOR CONTRIBUTIONS

All authors have made substantial contributions to this study. LMG, CR and SH were responsible for the conception and design of the study. LMG, CR, SH and AM developed the protocol and study approach. LMG drafted the manuscript. All authors read, revised and approved the final manuscript. LMG is guarantor.

ACKNOWLEDGEMENTS

Not applicable.

For peer review only - http://bmjopen.bmj.com/site/about/guidelines.xhtml

FIGURE LEGENDS

Figure 1. Medical Research Council's framework for the development and evaluation of complex interventions. Reproduced from Craig P. *et al.* British Medical Journal. 2008; 337:a1655.

Figure 2. Schematic diagram of Phase I: Development of HEAL-D using evidence synthesis and co-design methodology to design a culturally-tailored self-management programme for T2D in African and Caribbean communities

Figure 3. The Capability-Opportunity-Motivation (COM-B) Framework and Behaviour Change Wheel; a framework for developing behavioural interventions. Reproduced from Michie S., van Stralen M.M. and West R. Implementation Science. 2011; 6:42.

Figure 4. Applying the COM-B behaviour change framework to the development of the HEAL-D intervention; identifying theory of change.

For peer review only

For peer review only - http://bmjopen.bmj.com/site/about/guidelines.xhtml

Table 1. Topic guides for patient focus groups and stakeholder interviews

**Patient focus groups**	
	Knowledge and perceptions of diabetes, and diet and lifestyle advice for managing diabetes.
	Current practices relating to diabetes self-care, and diet and lifestyle.
	Health concerns/priorities in relation to diabetes.
	Motivations and barriers/difficulties relating to diabetes self-care, weight management and diet and lifestyle.
	Experiences and perceptions of diabetes care/education, and barriers to accessing care.
	Experiences of behaviour change in relation to diabetes, weight, diet and lifestyle – successes and failures.
	Role of family/friends/communities in influencing and shaping knowledge and behaviours in relation to diabetes, diet and lifestyle.
**Community leader interviews (including religious leaders)**	
	Health problems affecting the community and diabetes impact on health within this context.
	Attitude of the community towards health, medicines, doctors.
	Role of community leaders in promoting health and community activities.
	Diabetes health promotion activities within the community. What worked and what didn't.
	Barriers and facilitators to positive diabetes behaviours within the community.
	Advice about engaging the community: Who are the role models; What will engage and help people; How can healthcare & community work together.
**Healthcare professional interviews**	
	Experience of supporting African & Caribbean patients. What are the issues. How could things be improved. What factors make successful T2D management likely.
	Patient beliefs and motivations.
	Involvement in community activities and experience of working with community leaders and lay educators and suggestions to improve partnerships.
	Difficulties & challenges with offering a tailored lifestyle intervention.

For peer review only - http://bmjopen.bmj.com/site/about/guidelines.xhtml

Table 2. Mapping of the HEAL-D feasibility study research questions, process evaluation data sources and evaluation methods

PROCESS EVALUATION DOMAIN & RESEARCH QUESTIONS	DATA SOURCES: Patient questionnaires	Session observations	Session record of activities	Patient evaluation forms	Educator interviews	Patient interviews	Patient focus groups	Attendance records	HCP interviews	Commissioner interviews	EVALUATION METHOD
**TESTING INTERVENTION THEORY & MECHANISMS OF CHANGE**											
Are the intervention's mechanisms of change operationalised as hypothesised?	X	X	X	X	X	X	X				Qualitative data collected through interviews/focus groups with patients and educators, and session observation notes will be used to evaluate how the theory of the intervention operationalises and interacts with contextual factors.
How is the operationalisation of the mechanisms of change influenced by contextual factors?		X	X		X	X	X				
Does the interaction of the mechanisms of change with contextual factors give rise to unintended effects?		X	X		X	X	X				
**ASSESSING USUAL PRACTICE & CONTAMINATION**											
Is HEAL-D differentiable from 'usual practice'?						X					Interviews will be conducted with patients from both arms. Experiences of the intervention and control will be explored. With control patients issues of contamination and perceptions of 'usual care' will be discussed.
Is there contamination in control patients?						X					
**ASSESSING IMPLEMENTATION**											
What is the intervention reach and dose?	X							X			Questionnaire data will assess who receives the intervention and how representative they are e.g. age, gender, ethnicity, working status. Attendance records will be used to quantify the proportion of patients receiving the full *vs* part intervention.

For peer review only

For peer review only - http://bmjopen.bmj.com/site/about/guidelines.xhtml

Are the HEAL-D components/sessions delivered with fidelity and what is the nature of any adaptions?	X	X		X						To assess fidelity and compare intervention deliveries and contextual impacts educators will complete a record of activities & materials and list any resources/activities/discussions that were additional to the standardised schedule. These will be explored in depth in educator interviews which will be conducted at the end of the programme delivery. The research team will observe HEAL-D delivery to quantitatively assess coverage of curriculum, use of supporting materials and behaviour change techniques, quality of delivery, and participant engagement (binary score or a five-point Likert scale). Observers will qualitatively document course adaptations and general contextual observations.
Does the delivery of HEAL-D differ between sites, and what gives rise to differences?	X	X		X						
How well are the HEAL-D components/sessions delivered?	X	X								
**ASSESSING INTERVENTION ACCEPTABILITY**										
Is HEAL-D acceptable to patients, commissioners and healthcare professionals?	X		X		X	X		X	X	Acceptability will be evaluated through a range of qualitative and quantitative data. Quantitative data will be generated in patient evaluations, which will use 10-point scales to assess their views on the quality of the programme content, structure, format and delivery; the sessions/programme will be deemed 'acceptable' where they score ≥6 points. Interviews/focus groups with patients, educators, healthcare professionals and commissioners will explore acceptability through qualitative data e.g. reasons for attendance/non-attendance among patients, suggestions for amendments.
**ASSESSING INTERVENTION SUSTAINABILITY**										
How likely is the HEAL-D intervention to be sustainable and what factors might ensure sustainability?								X	X	Qualitative data collected through interviews with healthcare professionals and commissioners will be used to evaluate barriers & facilitators to implementation of HEAL-D into current care pathways, and its fit with organisational

For peer review only

		priorities, and the feasibility of sustained resource allocation to the HEAL-D intervention if found to be successful.

HCP, Healthcare professionals

For peer review only

For peer review only - http://bmjopen.bmj.com/site/about/guidelines.xhtml

REFERENCES

1. Estimating the current and future costs of Type 1 and Type 2 diabetes in the United Kingdom, including direct health costs and indirect societal and productivity costs [Internet]. York Health Economics Consortium Ltd. 2012.
2. Wilson C, Alam R, Latif S, Knighting K, Williamson S, Beaver K. Patient access to healthcare services and optimisation of self-management for ethnic minority populations living with diabetes: a systematic review. Health Soc Care Community. 2012;20(1):1-19.
3. Becker E, Boreham R, Chaudhury M, Craig R, Deverill C, Doyle M, et al. Health Survey for England 2004. The health of minority ethnic groups. London: Joint Health Surveys Unit, National Centre for Social Research, Department of Epidemiology and Public Health at the Royal Free and University College Medical School; 2006.
4. Paul SK, Owusu Adjah ES, Samanta M, Patel K, Bellary S, Hanif W, et al. Comparison of body mass index at diagnosis of diabetes in a multi-ethnic population: A case-control study with matched non-diabetic controls. Diabetes Obes Metab. 2017;19(7):1014-23.
5. Ng M, Fleming T, Robinson M, Thomson B, Graetz N, Margono C, et al. Global, regional, and national prevalence of overweight and obesity in children and adults during 1980-2013: a systematic analysis for the Global Burden of Disease Study 2013. Lancet. 2014;384(9945):766-81.
6. Lanting LC, Joung IM, Mackenbach JP, Lamberts SW, Bootsma AH. Ethnic differences in mortality, end-stage complications, and quality of care among diabetic patients: a review. Diabetes Care. 2005;28(9):2280-8.
7. James GD, Baker P, Badrick E, Mathur R, Hull S, Robson J. Type 2 diabetes: a cohort study of treatment, ethnic and social group influences on glycated haemoglobin. BMJ Open. 2012;2(5).
8. Healthcare for London. Diabetes guide for London. 2009.
9. London Assembly. Review of diabetes care in London. 2013.
10. ONS. Census data 2011. 2011.
11. Leung G, Stanner S. Diets of minority ethnic groups in the UK: influence on chronic disease risk and implications for prevention. British Nutrition Bulletin. 2011;36:161-98.
12. Department of Health. Improving quality of life for people with long term conditions. London, UK: Department of Health; 2013.
13. Department of H. National service framework for diabetes standards2001.
14. National Institute for Health and Care Excellence. Type 2 diabetes in adults: management (NG28). 2015.
15. National Institute for Health and Care Excellence Quality and Outcomes Framework indicator. MN27: The percentage of patients newly diagnosed with diabetes, on the register, in the preceding 1 April to 31 March who have a record of being referred to a structured education programme within 9 months after entry on to the diabetes register. 2011.
16. Lakhani M. No patient left behind: how can we ensure world class primary care for black and minority ethnic people? London: Department of Health; 2008.
17. Healthcare Commission. Managing diabetes: improving services for people with diabetes. 2007.
18. Brown K, Avis M, Hubbard M. Health beliefs of African-Caribbean people with type 2 diabetes: a qualitative study. Br J Gen Pract. 2007;57(539):461-9.
19. Sarkar U, Piette JD, Gonzales R, Lessler D, Chew LD, Reilly B, et al. Preferences for self-management support: findings from a survey of diabetes patients in safety-net health systems. Patient Educ Couns. 2008;70(1):102-10.
20. Stone MA. Making education in diabetes culturally appropriate for patients. Practice Nursing. 2006;17(12):5.
21. Cross T, Bazron B, Dennis K, Isaacs M. Towards a culturally competent system of care. Washington D.C.: Georgetown University Child Development Center, CASSP Technical Assistance Center; 1989.

For peer review only - http://bmjopen.bmj.com/site/about/guidelines.xhtml

22. Hawthorne K, Robles Y, Cannings-John R, Edwards AG. Culturally appropriate health education for type 2 diabetes mellitus in ethnic minority groups. Cochrane Database Syst Rev. 2008(3):CD006424.
23. Liu JJ, Davidson E, Bhopal RS, White M, Johnson MRD, Netto G, et al. Adapting health promotion interventions to meet the needs of ethnic minority groups: mixed-methods evidence synthesis. Health Technol Assess. 2012;16(44).
24. Attridge M, Creamer J, Ramsden M, Cannings-John R, Hawthorne K. Culturally appropriate health education for people in ethnic minority groups with type 2 diabetes mellitus. Cochrane Database of Systematic Reviews. 2014(9):CD006424.
25. Matsumoto D. Culture and Psychology. Pacific Grove, CA.: Brooks/Cole.; 1996.
26. Resnicow K, Baranowski T, Ahluwalia JS, Braithwaite RL. Cultural sensitivity in public health: defined and demystified. Ethn Dis. 1999;9(1):10-21.
27. Dyson PA, Kelly T, Deakin T, Duncan A, Frost G, Harrison Z, et al. Diabetes UK evidence-based nutrition guidelines for the prevention and management of diabetes. Diabet Med. 2011;28(11):1282-8.
28. Medical Research Council. Developing and evaluating complex interventions: new guidance. 2006.
29. Di Noia J, Furst G, Park K, Byrd-Bredbenner C. Designing culturally sensitive dietary interventions for African Americans: review and recommendations. Nutr Rev. 2013;71(4):224-38.
30. O'Mara-Eves A, Brunton G, McDaid D, Oliver S, Kavanagh J, Jamal F, et al. Community engagement to reduce inequalities in health: a systematic review, meta-analysis and economic analysis. Public Health Res. 2013;1(4).
31. Norris SL, Nichols PJ, Caspersen CJ, Glasgow RE, Engelgau MM, Jack L, et al. Increasing diabetes self-management education in community settings. A systematic review. Am J Prev Med. 2002;22(4 Suppl):39-66.
32. National Institute for Health and Care Excellence. Behaviour change: individual approaches. 2014. Report No.: Public health guidelines (PH49).
33. Osei-Assibey G, Boachie C. Dietary interventions for weight loss and cardiovascular risk reduction in people of African ancestry (blacks): a systematic review. Public Health Nutr. 2012;15(1):110-5.
34. Wolfe WA. A review : maximizing social support-a neglected strategy for improving weight management with African-American women. Ethn Dis. 2004;14:212-8.
35. Kumanyika SK, Wadden TA, Shults J, et al. Trial of family and friend support for weight loss in african american adults. Archives of Internal Medicine. 2009;169(19):1795-804.
36. Sumlin LL, Garcia AA. Effects of food-related interventions for African American women with type 2 diabetes. Diabetes Educ. 2012;38(2):236-49.
37. Samuel-Hodge CD, DeVellis RF, Ammerman A, Keyserling TC, Elasy TA. Reliability and validity of a measure of perceived diabetes and dietary competence in African American women with type 2 diabetes. Diabetes Educ. 2002;28(6):979-88.
38. Abubakari AR, Jones MC, Lauder W, Kirk A, Anderson J, Devendra D, et al. Ethnic differences and socio-demographic predictors of illness perceptions, self-management, and metabolic control of type 2 diabetes. Int J Gen Med. 2013;6:617-28.
39. Michie S. Designing and implementing behaviour change interventions to improve population health. J Health Serv Res Policy. 2008;13 Suppl 3:64-9.
40. Quandt SA, Ip EH, Kirk JK, Saldana S, Chen SH, Nguyen H, et al. Assessment of a short diabetes knowledge instrument for older and minority adults. Diabetes Educ. 2014;40(1):68-76.
41. Anderson RM, Fitzgerald JT, Gruppen LD, Funnell MM, Oh MS. The Diabetes Empowerment Scale-Short Form (DES-SF). Diabetes Care. 2003;26(5):1641-2.
42. Zimet GD, Dahlem NW, Zimet SG, Farley GK. The Multidimensional Scale of Perceived Social Support. Journal of Personality Assessment. 2010;52(1):30-41.

For peer review only

For peer review only - http://bmjopen.bmj.com/site/about/guidelines.xhtml

43. McGuire BE, Morrison TG, Hermanns N, Skovlund S, Eldrup E, Gagliardino J, et al. Short-form measures of diabetes-related emotional distress: the Problem Areas in Diabetes Scale (PAID)-5 and PAID-1. Diabetologia. 2010;53(1):66-9.
44. Rabin R, de Charro F. EQ-5D: a measure of health status from the EuroQol Group. Ann Med. 2001;33(5):337-43.
45. Moore GF, Audrey S, Barker M, Bond L, Bonell C, Hardeman W, et al. Process evaluation of complex interventions: Medical Research Council guidance. BMJ. 2015;350:h1258.

For peer review only

For peer review only - http://bmjopen.bmj.com/site/about/guidelines.xhtml

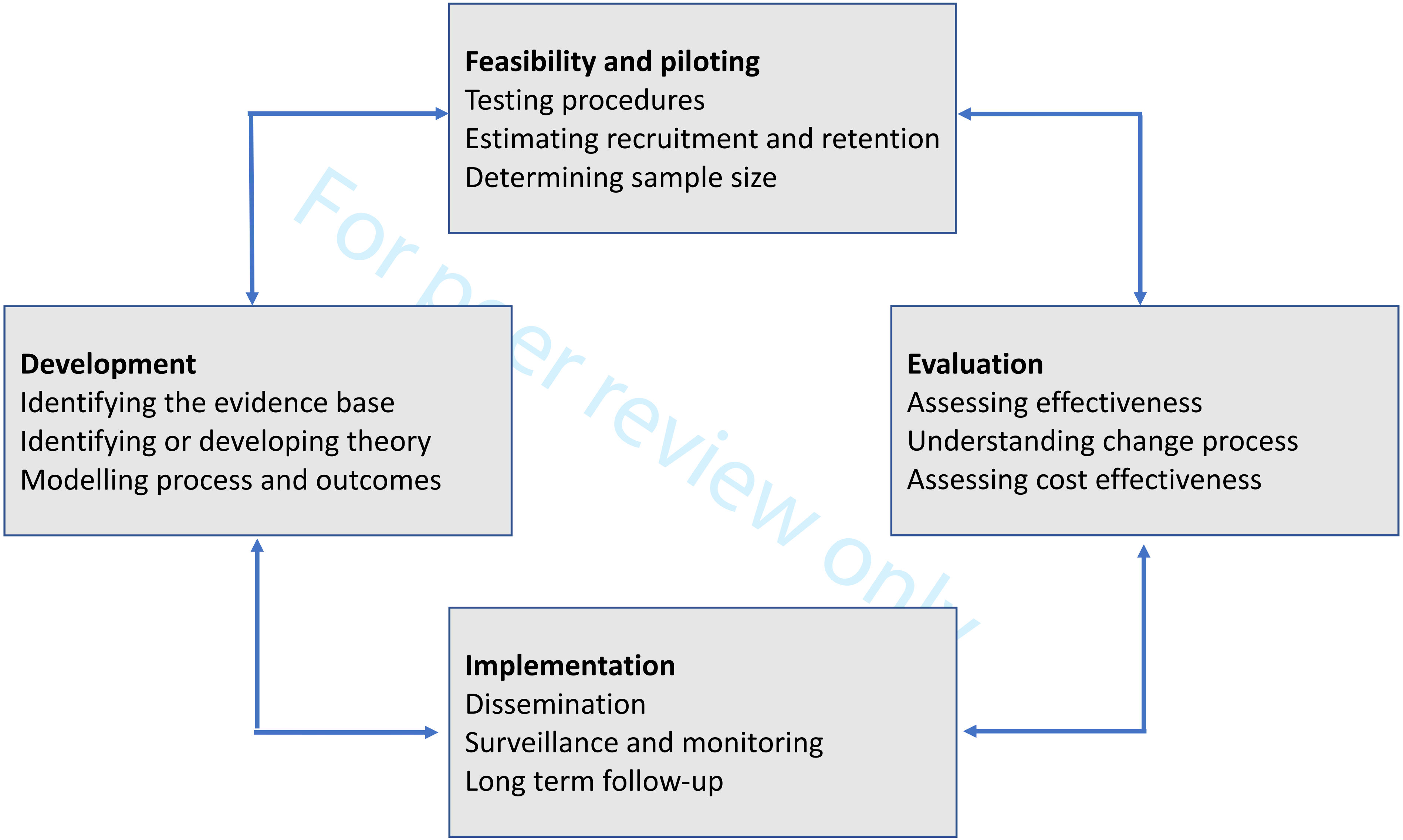

For peer review only - http://bmjopen.bmj.com/site/about/guidelines.xhtml

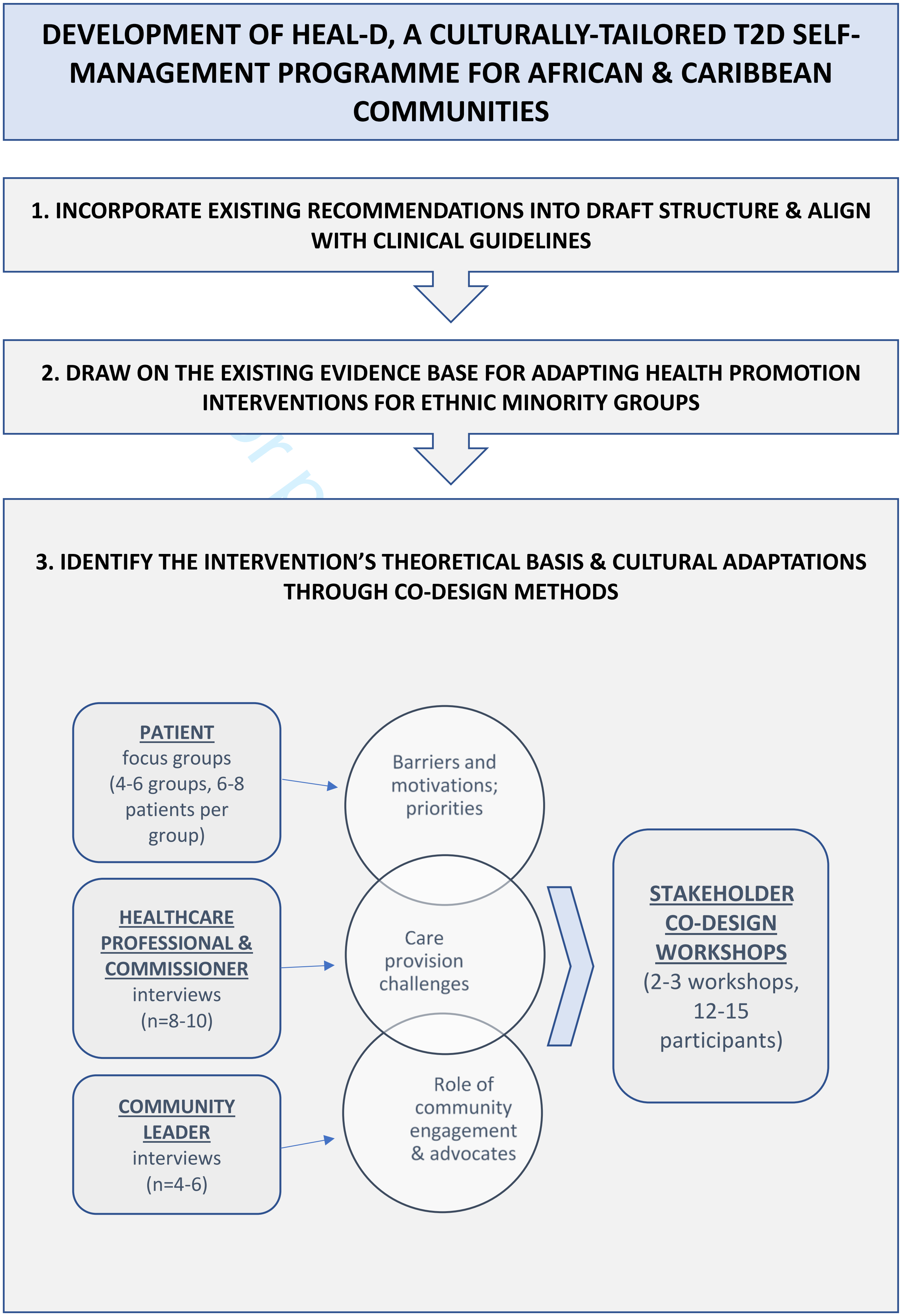
DEVELOPMENT OF HEAL-D, A CULTURALLY-TAILORED T2D SELF-MANAGEMENT PROGRAMME FOR AFRICAN & CARIBBEAN COMMUNITIES
1. INCORPORATE EXISTING RECOMMENDATIONS INTO DRAFT STRUCTURE & ALIGN WITH CLINICAL GUIDELINES
2. DRAW ON THE EXISTING EVIDENCE BASE FOR ADAPTING HEALTH PROMOTION INTERVENTIONS FOR ETHNIC MINORITY GROUPS
3. IDENTIFY THE INTERVENTION'S THEORETICAL BASIS & CULTURAL ADAPTATIONS THROUGH CO-DESIGN METHODS
PATIENT focus groups (4-6 groups, 6-8 patients per group)
HEALTHCARE PROFESSIONAL & COMMISSIONER interviews (n=8-10)
COMMUNITY LEADER interviews (n=4-6)
Barriers and motivations; priorities
Care provision challenges
Role of community engagement & advocates
STAKEHOLDER CO-DESIGN WORKSHOPS (2-3 workshops, 12-15 participants)

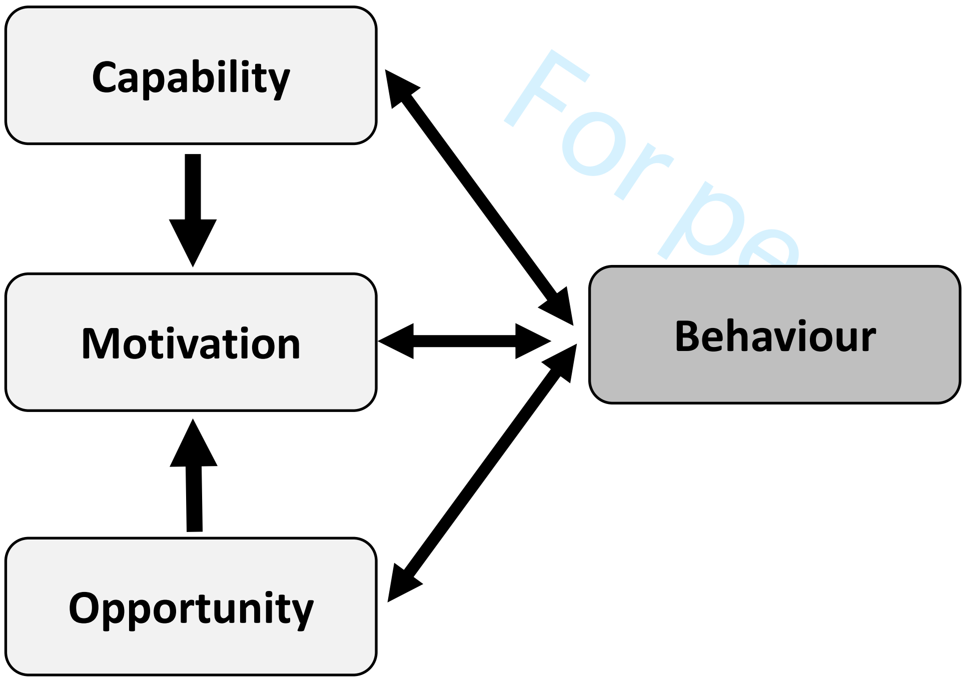
Capability
Motivation
Opportunity
Behaviour

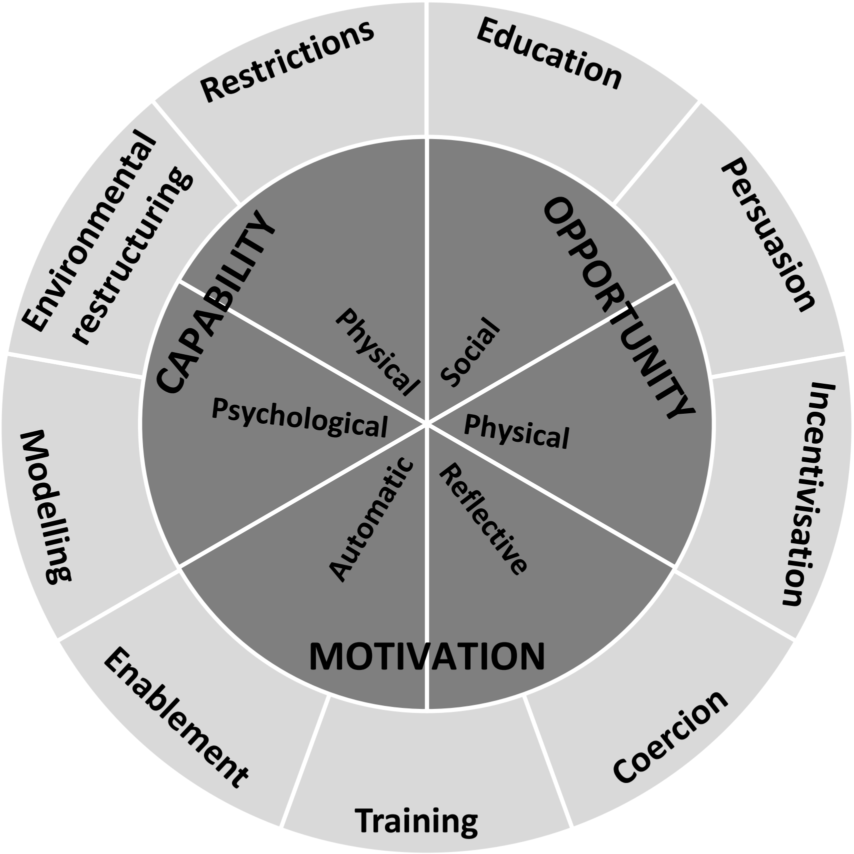
Restrictions
Education
Persuasion
Incentivisation
Coercion
Training
Enablement
Modelling
Environmental restructuring
CAPABILITY
OPPORTUNITY
MOTIVATION
Physical
Psychological
Social
Physical
Automatic
Reflective

COM-B analysis of the behaviour goal: performing 30 minutes moderate intensity physical activity per day

CAPABILITY

Knowledge: Does the target group know:

-Why moderate intensity physical activity would help diabetes management?

-What moderate intensity is and how to measure it?

Behavioural regulation: Does the target group know how to:

-Plan to fit the activity in to their daily life?

-Remember to do the activity?

-Prioritise this activity over others?

-Record & measure and self-monitor their activity?

Physical skills: Does the target group:

Have the physical stamina to be active at this intensity?

OPPORTUNITY

Environmental context & resources:

-Is it safe to exercise in the neighbourhood?

-Do patients have suitable footwear?

-Can they afford a pedometer or some means of measuring their activity?

Social influences (what interpersonal influences cause individuals to change their thoughts, feelings or behaviours?)

-It is culturally acceptable to exercise?

-What is the social norm among immediate friends and family?

-What positive or negative views do others have that may influence activity?

-Are there any positive role models?

-Are there competing demands e.g. pressure to spend leisure time with family or at church?

Would group support be motivating?

MOTIVATION

Reflective (conscious) motivation:

-How optimistic do the patients feel they can achieve the goal?

-Do they intend to do the behaviour (stages of change model)?

-What emotions may help or hinder? e.g. fear of injury.

-What other emotions may conflict? e.g tiredness, depression, stress.

Automatic (innate drivers):

-What are established habit patterns?

-What are routines/thought/behaviours set up by previous experience
